# Widespread Adaptive Introgression of Major Histocompatibility Complex Genes across Vertebrate Hybrid Zones

**DOI:** 10.1093/molbev/msae201

**Published:** 2024-09-26

**Authors:** T Gaczorek, K Dudek, U Fritz, L Bahri-Sfar, S J E Baird, F Bonhomme, C Dufresnes, V Gvoždík, D Irwin, P Kotlík, S Marková, P McGinnity, M Migalska, J Moravec, L Natola, M Pabijan, K P Phillips, Y Schöneberg, A Souissi, J Radwan, W Babik

**Affiliations:** Institute of Environmental Sciences, Faculty of Biology, Jagiellonian University, Kraków, Poland; Institute of Environmental Sciences, Faculty of Biology, Jagiellonian University, Kraków, Poland; Museum of Zoology (Museum für Tierkunde), Senckenberg Dresden, Dresden, Germany; Biodiversité, Parasitologie et Ecologie des Ecosystèmes Aquatiques, Faculté des Sciences de Tunis, Univ de Tunis El Manar, Tunis, Tunisia; Institute of Vertebrate Biology of the Czech Academy of Sciences, Brno, Czech Republic; Institut des Sciences de l'Evolution, Université de Montpellier, Montpellier, France; Institut de Systématique, Evolution, Biodiversité (ISYEB), Muséum National d’Histoire Naturelle, CNRS, Sorbonne Université, EPHE, Université des Antilles, Paris, France; Institute of Vertebrate Biology of the Czech Academy of Sciences, Brno, Czech Republic; Department of Zoology, National Museum of the Czech Republic, Prague, Czech Republic; Biodiversity Research Centre and Department of Zoology, University of British Columbia, Vancouver, British Columbia, Canada; Laboratory of Molecular Ecology, Institute of Animal Physiology and Genetics of the Czech Academy of Sciences, Liběchov, Czech Republic; Laboratory of Molecular Ecology, Institute of Animal Physiology and Genetics of the Czech Academy of Sciences, Liběchov, Czech Republic; School of Biological, Earth and Environmental Sciences, University College Cork, Cork, Ireland; Institute of Environmental Sciences, Faculty of Biology, Jagiellonian University, Kraków, Poland; Department of Zoology, National Museum of the Czech Republic, Prague, Czech Republic; Biodiversity Research Centre and Department of Zoology, University of British Columbia, Vancouver, British Columbia, Canada; Institute of Zoology and Biomedical Research, Faculty of Biology, Jagiellonian University, Kraków, Poland; Laboratory of Molecular Ecology, Institute of Animal Physiology and Genetics of the Czech Academy of Sciences, Liběchov, Czech Republic; Canadian Rivers Institute, University of New Brunswick, Fredericton, New Brunswick, Canada; Senckenberg Biodiversity and Climate Research Centre (BiK-F), Frankfurt am Main, Germany; Institute for Ecology, Evolution and Diversity, Goethe University, Frankfurt am Main, Germany; Biodiversité, Parasitologie et Ecologie des Ecosystèmes Aquatiques, Faculté des Sciences de Tunis, Univ de Tunis El Manar, Tunis, Tunisia; MARBEC, Univ Montpellier, 34000 Montpellier, France; Institute of Environmental Biology, Faculty of Biology, Adam Mickiewicz University, Poznań, Poland; Institute of Environmental Sciences, Faculty of Biology, Jagiellonian University, Kraków, Poland

**Keywords:** adaptation, host–pathogen coevolution, hybridization, introgression, MHC

## Abstract

Interspecific introgression is a potentially important source of novel variation of adaptive significance. Although multiple cases of adaptive introgression are well documented, broader generalizations about its targets and mechanisms are lacking. Multiallelic balancing selection, particularly when acting through rare allele advantage, is an evolutionary mechanism expected to favor adaptive introgression. This is because introgressed alleles are likely to confer an immediate selective advantage, facilitating their establishment in the recipient species even in the face of strong genomic barriers to introgression. Vertebrate major histocompatibility complex genes are well-established targets of long-term multiallelic balancing selection, so widespread adaptive major histocompatibility complex introgression is expected. Here, we evaluate this hypothesis using data from 29 hybrid zones formed by fish, amphibians, squamates, turtles, birds, and mammals at advanced stages of speciation. The key prediction of more extensive major histocompatibility complex introgression compared to genome-wide introgression was tested with three complementary statistical approaches. We found evidence for widespread adaptive introgression of major histocompatibility complex genes, providing a link between the process of adaptive introgression and an underlying mechanism. Our work identifies major histocompatibility complex introgression as a general mechanism by which species can acquire novel, and possibly regain previously lost, variation that may enhance defense against pathogens and increase adaptive potential.

## Introduction

The process of adaptation by natural selection requires genetic variation within populations. Consequently, the rate and extent of adaptation may be limited by the availability of suitable variation (Bell 2013; Rousselle et al. 2020). One potentially important source of such variation is introgression, which is the acquisition of new genetic variants through hybridization with closely related species (Edelman and Mallet 2021; Moran et al. 2021). Any introgressed variants will have been pretested by natural selection. Moreover, their initial frequencies in the recipient species are often higher than those of variants produced by de novo mutation, which may facilitate adaptation by reducing the risk of stochastic loss during the establishment phase (Hedrick 2013). While reproductive barriers and reduced fitness of hybrids limit introgression (Barton and Bengtsson 1986; Nosil et al. 2017; Butlin and Smadja 2018; Barton 2020), both theory (Barton 1979; Piálek and Barton 1997) and accumulating empirical evidence (Hedrick 2013; Enard and Petrov 2018; Edelman and Mallet 2021; Moran et al. 2021) indicate that genome-wide barriers to introgression can be readily overcome by genetic variants that confer a selective advantage in the recipient species. Nonetheless, empirically supported, process-based generalizations about the targets and mechanisms of adaptive introgression are lacking.

Certain functional categories of genes, such as those involved in immunity (Enard and Petrov 2018), pigmentation (Giska et al. 2019), or resistance to environmental (Whitney et al. 2006) and anthropogenic (Song et al. 2011) stressors, appear to be more prone to adaptive introgression, possibly due to similar selective pressures experienced by related species. One evolutionary process that should particularly favor introgression is balancing selection, especially when operating through the mechanism of rare allele advantage, which maintains multiple alleles in a population (multiallelic balancing selection) via negative frequency dependence (Castric et al. 2008; Fijarczyk and Babik 2015). Because introgressed alleles are initially rare, they would often confer a selective advantage, facilitating their establishment in the recipient species even in the face of strong genomic barriers to introgression, while negative frequency dependence will prevent their fixation (Castric et al. 2008; Fijarczyk et al. 2018).

A prime example of genes evolving under long-term balancing selection is the major histocompatibility complex (MHC) in jawed vertebrates (Spurgin and Richardson 2010). The MHC encodes two classes of molecules—Class I (MHC-I) and Class II (MHC-II), both of which present antigens to T cells, but differ in, among other features, the source of antigenic peptides and the subpopulations of T cells with which they interact (Murphy et al. 2022). Because of their key role in initiating the adaptive immune response, both MHC classes are involved in perpetual Red Queen coevolutionary dynamics with pathogens (Baird et al. 2012; Radwan et al. 2020). An advantage of MHC alleles from allopatric populations (novel to local parasites) has been both predicted (Baird et al. 2012) and experimentally demonstrated (Phillips et al. 2018). There is also evidence for MHC introgression, possibly adaptive, in several systems, including humans (Abi-Rached et al. 2011), goats (Grossen et al. 2014), and newts (Dudek et al. 2019). If our understanding of the mechanisms promoting introgression and maintaining MHC diversity is correct, taxonomically widespread adaptive MHC introgression is expected, and this prediction can be tested in a comparative framework. However, testing for adaptive introgression of genes under balancing selection in general, and the MHC in particular, presents significant challenges. First, balancing selection itself may generate signatures similar to the signal of introgression in a way that confounds testing by, e.g. long-term maintenance of shared polymorphism (Fijarczyk and Babik 2015). Second, the genomic organization of the MHC region is often extremely complex (McConnell et al. 2016; Westerdahl et al. 2022), with multiple similar, highly variable genes exhibiting extensive intraspecific copy number variation. The latter makes the assembly of the MHC region difficult and its representation by a single reference sequence often impossible (Dilthey 2021), which in turn precludes the use of computational methods designed to detect adaptive introgression from genome resequencing data (Setter et al. 2020; Gower et al. 2021; Svedberg et al. 2021). Third, the dynamic evolution of the MHC region involves frequent duplication, pseudogenization and gene losses, as well as interlocus recombination. Combined with considerable allelic divergence within MHC loci, these processes produce patterns of sequence divergence that often preclude reliable assignment of MHC variants to loci. This is particularly evident for the data typically used to characterize MHC variation in natural populations—sequences of PCR-amplified fragments of variable exons. Given the nature of the data, it is necessary to employ analytical methods that do not rely on the assignment of MHC variants to loci.

Direct comparison of the extent of MHC introgression with that of genome-wide introgression in natural hybrid zones, applied to the multilocus MHC data, may help to overcome the challenges outlined above (Payseur 2010; Gompert et al. 2017; Dudek et al. 2019). For example, shared ancestral polymorphism would be expected to be uniformly distributed across species ranges, whereas introgressing variants should initially be concentrated near the hybrid zone, resulting in locally increased interspecific similarity. Recent or ongoing adaptive introgression would generate a signal of similarity extending further from the hybrid zone than that of genome-wide, neutral introgression. Here, we use data from 29 hybrid zones formed by fish, amphibians, squamates, turtles, birds, and mammals and employ three complementary statistical approaches to compare MHC introgression with genome-wide introgression. We synthesize the results in a comparative framework and provide evidence for widespread adaptive introgression of MHC genes across vertebrate hybrid zones, which leads to an important generalization about the process.

## Results

**Fig. 1.**
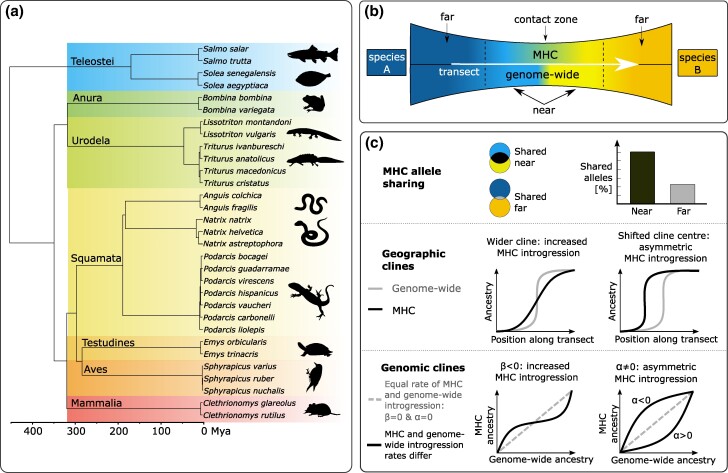
Study design. a) Time-calibrated phylogeny of the species studied; b) the geographic extent of genome wide and MHC introgression expected under adaptive MHC introgression, along with the division of species ranges into areas classified as “near” and “far” from the contact zone; c) the approaches used to compare genome-wide and MHC introgression.

We examined 29 hybrid zones formed by 31 species representing 11 genera of seven major vertebrate lineages (Fig. 1a); a total of 4,933 individuals were analyzed (supplementary table S1, Supplementary Material online). The selection of hybrid zones was based on the following criteria: (i) the taxa were known to have limited genome-wide introgression (see Genome-Wide Admixture)—which was crucial for our tests, which detect signals of MHC introgression extending beyond the geographic range of genome-wide introgression; (ii) reliable, individual-based genome-wide estimates of interspecific admixture were available; and (iii) DNA or tissue samples for MHC genotyping were accessible.

### MHC Diversity

MHC data for eight genera were generated for the purposes of this study, while for the remaining three genera, data were derived entirely (Gaczorek et al. 2023a, 2023b) or partially (Dudek et al. 2019) from previous work. MHC diversity was assessed by Illumina sequencing of the PCR-amplified second exon of MHC-I and MHC-IIB genes. This exon is part of the peptide-binding region that determines the antigen specificity of MHC molecules and is highly variable. For simplicity, we refer to each MHC sequence variant as an allele, although we are aware that these variants are derived from multiple loci and hence an individual typically carries more than two such defined MHC alleles. The repeatability of genotyping, estimated by running a fraction of the samples in duplicate, averaged across genera was 0.95 (range 0.88 to 1.00) for MHC-I and 0.91 (range 0.76 to 1.00) for MHC-II (supplementary table S2, Supplementary Material online).

**Fig. 2.**
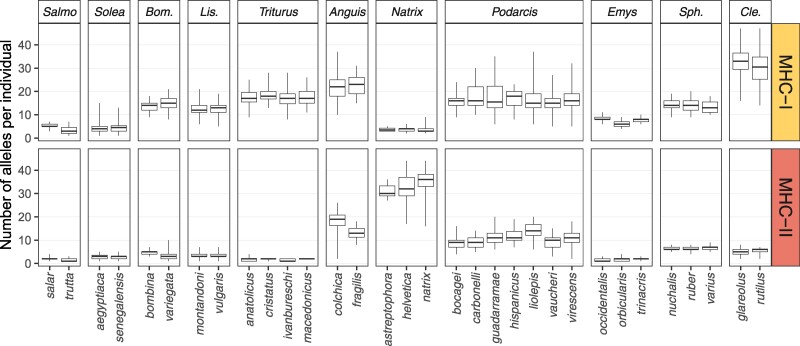
The number of MHC alleles per individual in the studied species. These results show clear differences in the complexity between MHC classes and between taxa. Note that we use term “allele” for each unique sequence variant, although these variants may originate from different MHC genes. Boxplots show the median (horizontal line), the first and third quartile (box), and the range (whiskers). Different MHC markers were used for *Clethrionomys* (see Materials and Methods), so estimates of MHC variation for this genus are not directly comparable with those for other taxa. *Bom.*, *Bombina*; *Lis.*, *Lissotriton*; *Sph.*, *Sphyrapicus*; *Cle.*, *Clethrionomys*.

The range of MHC variation, measured as both the total and sample size standardized number of alleles per species, i.e. allelic richness, spanned two orders of magnitude (supplementary table S3, Supplementary Material online). In all genera except *Natrix*, MHC-I was more diverse than MHC-II. The mean number of alleles per individual was similar for species within a genus but varied more than tenfold between genera, ranging from 3.7 (*Natrix*) to 33.7 (*Clethrionomys*) in MHC-I and from 1.8 (*Triturus*) to 34.6 (*Natrix*) in MHC-II (Fig. 2). The number of alleles per individual may be indicative of the number of MHC genes—the minimum number of genes, assuming all were heterozygous, would be half the number of alleles. As almost all taxa had more than one gene per each MHC class and unambiguous assignment of alleles to specific genes was not possible, MHC genotypes were coded as binary matrices of allele presence/absence. These matrices were used in subsequent analyses of introgression. For each hybrid zone, the number of rows in the binary genotype matrix was equal to the number of individuals examined, and the number of columns was equal to the total number of detected alleles, with a “1” at the intersection of a column and a row indicating the presence of the allele in the individual and “0” indicating its absence.

### Comparison of MHC and Genome-Wide Introgression

We tested two major patterns predicted by the adaptive MHC introgression hypothesis for hybrid zones—the theoretical expectations are summarized in Fig. 1b and c. First, MHC introgression should extend geographically further than genome-wide introgression. This prediction is based on two assumptions: (i) selective advantage allows introgressed MHC alleles to increase in frequency and rapidly spread outside the contact zone, and (ii) the perpetual nature of the host–pathogen arms race sustains an influx of foreign MHC alleles over extended periods of time, resulting in a broad MHC introgression gradient (Fig. 1b). We tested this prediction using two approaches (Fig. 1c): (i) randomizations to test for elevated interspecific MHC allele sharing in the vicinity of contact zones (near) but beyond the actual hybrid zone (defined as the area of genome-wide introgression detectable using single nucleotide polymorphism (SNP) or microsatellite markers), compared to the areas further from the contact (far), and (ii) comparison of the width and position of MHC and genome-wide geographic clines, which measure the transition in population-level ancestry of each species along a geographic transect. The second prediction is a relative excess of heterospecific ancestry in MHC compared to the genome-wide average in admixed individuals, which would indicate a selective advantage of heterospecific MHC even in early generation hybrids. This prediction was tested using the genomic clines approach (Fig. 1c).

#### MHC Allele Sharing

**Fig. 3.**
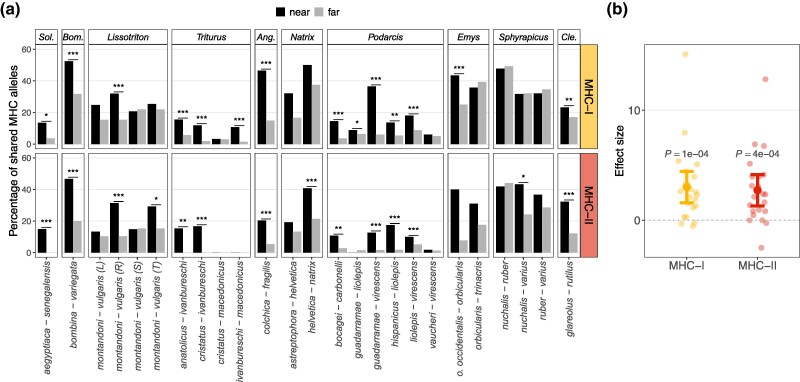
Interspecific MHC allele sharing was elevated in the vicinity of contact but beyond the actual hybrid zone (“near”) compared to the areas further from the contact (“far”). a) Allele sharing between species near and far from the contact zone; b) SESs (Cohen's *d*) of the difference in allele sharing between species near and far from the contact zone and the PGLS model estimates with 95% confidence intervals; *Sol.*, *Solea*; *Bom.*, *Bombina*; *Ang.*, *Anguis*; *Cle.*, *Clethrionomys*.

The significance of differences in allele sharing between species near and far from the contact zone was tested with a permutation test, randomly assigning individuals within species into near/far categories. Randomization tests were performed in 25 hybrid zones representing ten genera (supplementary table S4, Supplementary Material online). In most cases, the proportion of alleles shared between species was higher near than far from the contact, the difference being significant for at least one MHC class in 17 out of 25 (68%) zones and for both MHC classes in 11 out of 25 (44%) zones (Fig. 3a). In addition, in *Salmo*, we detected significantly increased allele sharing within the contact zone for both MHC classes (*P* < 0.001), which is indicative of MHC introgression but is not informative about its adaptiveness due to the lack of the near category. The results were summarized using standardized effect size (SES; Gotelli and McCabe 2002; Botta-Dukát 2018), which allowed a meaningful comparison of the excess of MHC allele sharing near the contact zone across the investigated taxa. Phylogenetic generalized least squares (PGLS) was used to assess the significance of SES. The phylogenetic correlation matrix input to PGLS accounted for the fact that our data were from pairs of species rather than single species. The SES was significantly greater than 0 for both MHC classes (MHC-I *P* = 0.0001, MHC-II *P* = 0.0004; Fig. 3b), indicating elevated allele sharing near the contact, with no significant difference between MHC classes (*P* = 0.78). When time of divergence between hybridizing species was included in the PGLS model (supplementary fig. S1a, Supplementary Material online), its effect was not significant (*P* = 0.42, model without class × time interaction); including interaction did not improve model fit (interaction likelihood ratio test [LRT]; *P* = 0.74). There was no phylogenetic signal in either model (Pagel's *λ* = 0.0).

#### Geographic Clines

Transects were designated for 19 hybrid zones representing eight genera. Geographic clines could be fitted to all transects except those for *Natrix astreptophora* × *N. helvetica* and one of the *N. helvetica* × *N. natrix* transects (HN3). In these two cases, a null model of no cline (i.e. straight line) was supported for at least one MHC class and these transects were excluded from the comparative analysis of geographic clines, as the values of cline parameters would not be interpretable.

The use of binary-coded MHC genotypes to calculate the hybrid index (HI), which is then used to fit clines, has the potential to affect the accuracy of cline parameter estimates. To explore whether this was the case, we simulated five-locus haplotypes evolving under strong negative frequency-dependent selection in two species that evolved in isolation following divergence from a common ancestor. At three time points, 0.5, 1, and 4N generations after divergence, we created a “transect” through a hybrid zone by sampling different proportions of MHC haplotypes from each species along a predefined smooth ancestry cline. We then estimated the cline width using: (i) the actual, known ancestry of the haplotypes; (ii) the hybrid index estimated from known genotypes at each MHC locus; and (iii) the hybrid index estimated considering each MHC allele as an independent binary dominant locus, reflecting the treatment of the observed MHC data. We found that binary coding did not affect the estimated cline width (supplementary fig. S2, Supplementary Material online), consistent with previous work that used a somewhat different simulation setup (Gaczorek et al. 2023b).

**Fig. 4.**
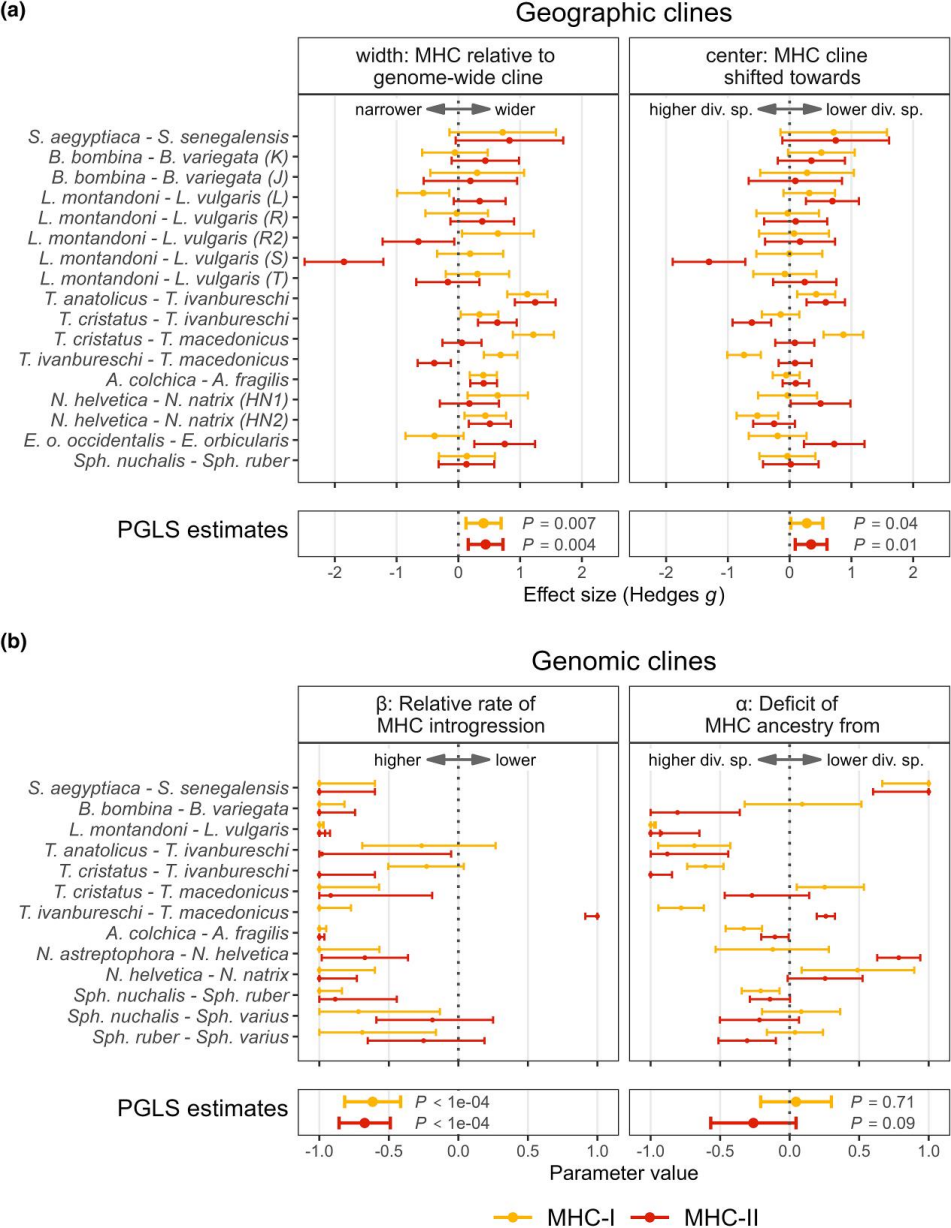
Comparison of MHC and genome-wide a) geographic and b) genomic clines indicates more extensive introgression of both MHC classes compared to the genome-wide introgression. For each hybrid zone and MHC class, points show effect size estimates with 95% confidence intervals (a) or the ML parameter estimates with 2 log-likelihood ranges (b). *S.*, *Solea*; *B.*, *Bombina*; *L.*, *Lissotriton*; *T.*, *Triturus*; *A.*, *Anguis*; *N.*, *Natrix*; *Sph.*, *Sphyrapicus*; PGLS estimates, phylogenetic generalized least squares estimates together with 95% confidence intervals; higher/lower div. sp., species in a pair exhibiting higher/lower MHC diversity. Note that uncertainty in parameter estimates for individual taxa is incorporated as weights in PGLS models, so confidence intervals of PGLS estimates should not be interpreted in the context of uncertainty in individual estimates.

**Fig. 5.**
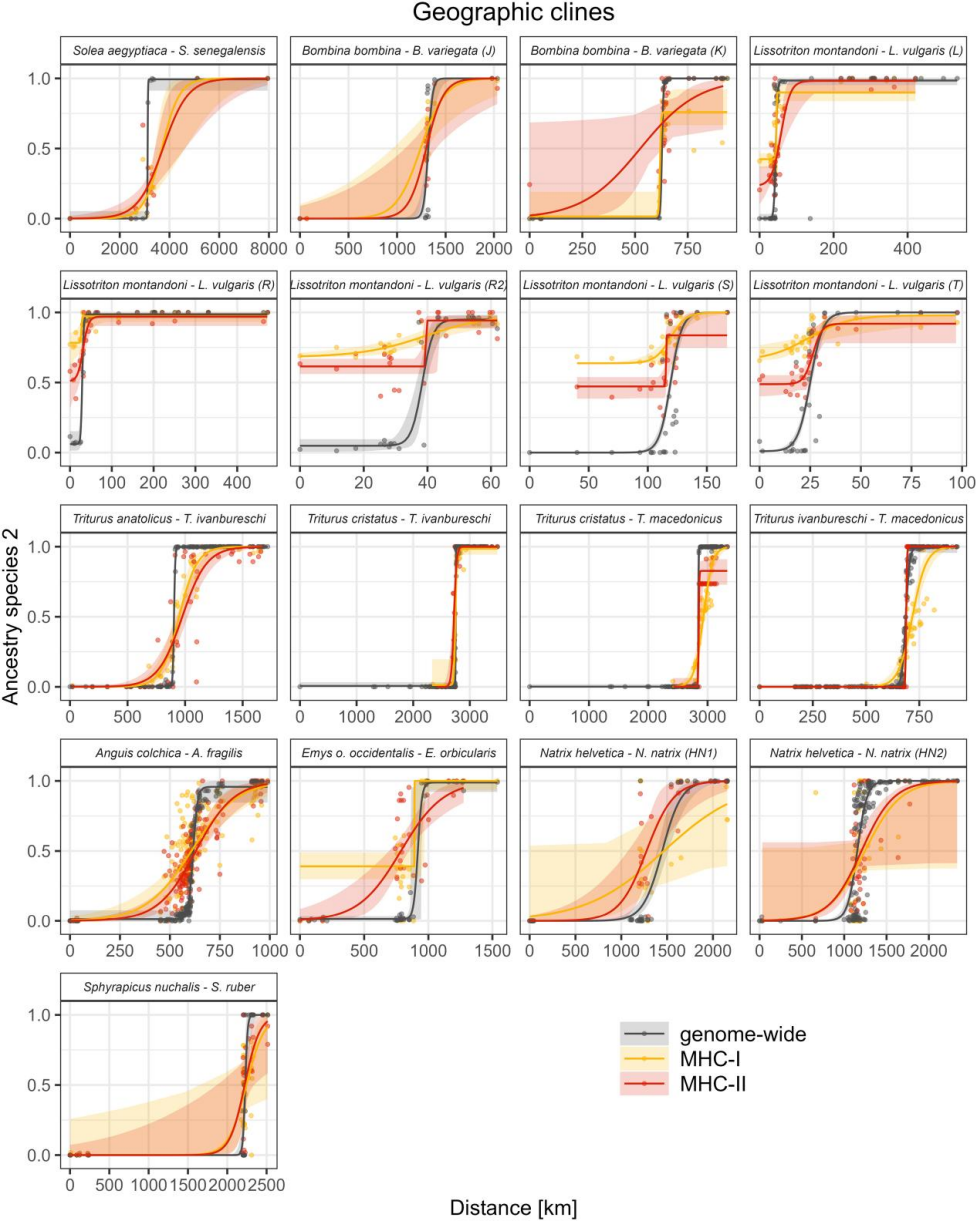
Comparison of the MHC and genome-wide geographic clines. Light-colored bands show 2 log-likelihood intervals, while points correspond to populations.

MHC clines tended to be wider than genome-wide clines. In all transects, a cline estimated for at least one MHC class was wider than the genome-wide cline, and in 9 out of 17 (53%) transects, clines for both MHC classes were wider (Figs. 4a and 5). In many cases, the 95% confidence intervals of SES were wide and overlapped 0, indicating no significant difference between MHC and genome-wide cline widths. This lack of significance was a direct consequence of the wide 2 log-likelihood intervals for the parameters of the MHC clines. The PGLS analysis of SES supported overall wider MHC clines (MHC-I *P* = 0.007, MHC-II *P* = 0.004; Fig. 4a) and no difference between classes (*P* = 0.86). In the model including time of divergence between hybridizing taxa (supplementary fig. S1b, Supplementary Material online), the effect of time was not significant (PGLS, *P* = 0.08, model without interaction, interaction LRT *P* = 0.48). There was no evidence of phylogenetic signal (*λ* = 0.0).

We found a significant MHC cline shift toward species with lower MHC diversity (PGLS, MHC-I *P* = 0.037, MHC-II *P* = 0.011; Fig. 4a) with no significant difference between classes (*P* = 0.70). In models including divergence time (supplementary fig. S1b, Supplementary Material online), its effect was not significant (PGLS, *P* = 0.24, model without interaction, interaction LRT *P* = 0.56). Again, there was no evidence of a phylogenetic signal (*λ* = 0.0).

#### Genomic Clines

**Fig. 6.**
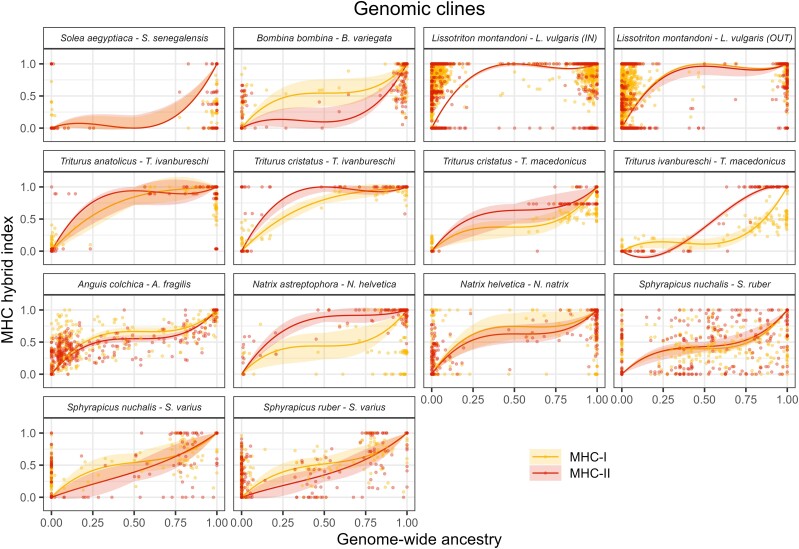
Genomic clines. Light-colored bands show 2 log-likelihood intervals, while points correspond to individuals.

We fitted genomic clines to data from 14 hybrid zones representing seven genera. In the other systems, the distribution of genome-wide ancestry was strongly bimodal, indicating very limited genome-wide introgression and precluding the use of genomic clines, which rely on comparing locus-specific and genome-wide ancestry in admixed individuals (Gompert and Buerkle 2011; Fitzpatrick 2013). The rate of MHC introgression exceeded that of genome-wide introgression (indicated by the negative value of the β parameter of genomic clines, reflecting the lower rate of transition in MHC ancestry compared to genome-wide ancestry) in all zones except MHC II in *Triturus ivanbureschi* × *Triturus macedonicus* (Figs. 4b and 6). The PGLS analysis supported a higher rate of MHC introgression compared to genome-wide introgression (*P* < 0.0001 for both MHC classes; supplementary fig. S1c, Supplementary Material online) and revealed no difference between MHC classes (*P* = 0.67). In the model including divergence time (supplementary fig. S1c, Supplementary Material online), there was a tendency for an increased rate of introgression between more distantly related species (PGLS, *P* = 0.04, model without interaction, interaction LRT *P* = 0.09), and no phylogenetic signal (*λ* = 0.0) was detected.

We did not observe an excess of MHC ancestry from the species with higher MHC variability (i.e. introgression toward species with lower MHC diversity, *α* > 0; Fig. 4b). The PGLS analysis did not support asymmetry, as *α* was not significantly different from 0 for either MHC class (MHC-I *P* = 0.71, MHC-II *P* = 0.09; Fig. 4b), and there was no significant difference between classes (*P* = 0.12). In the model including divergence time (supplementary fig. S1c, Supplementary Material online), there was a tendency toward higher values of *α* for more distantly related species (PGLS, *P* = 0.02, model without interaction, interaction LRT *P* = 0.71). There was no phylogenetic signal in models without time of divergence (*λ* = 0.0) while a substantial phylogenetic signal was detected in models including time of divergence (*λ* = 0.54).

## Discussion

Our results indicate that MHC introgression across vertebrate hybrid zones is more extensive than genome-wide introgression, strongly suggesting its adaptive nature. This finding is consistent with the expectation that rare allele advantage, a mechanism of balancing selection often acting on MHC genes as a consequence of coevolution with pathogens, should initially favor novel introgressed MHC variants (Radwan et al. 2020). Alleles that have already been tested for functionality in another species, but to which local pathogens are not yet adapted, may be particularly advantageous and thus introgress easily. A similar magnitude of introgression observed for both MHC classes suggests that such an advantage applies to both groups of these antigen-presenting molecules, despite evident functional differences between them (Pishesha et al. 2022).

The empirical results presented here support theoretical predictions (Schierup et al. 2000) of widespread adaptive introgression of genes evolving under balancing selection, offering a link between the process and the underlying mechanism. We expect adaptive introgression to be widespread also in other genetic systems evolving under long-term multiallelic balancing selection, such as self-incompatibility genes in plants, where it has indeed been documented in some taxa (Castric et al. 2008). Adaptive MHC introgression may be further facilitated by the fact that introgressed haplotypes would carry rare alleles of multiple MHC genes, thereby increasing the cumulative selective advantage. This effect could be amplified if the mechanism of divergent allele advantage, for which there is some empirical support (Pierini and Lenz 2018), was to operate. A strong fitness advantage of introgressed MHC haplotypes could help to overcome barriers to introgression due to negative epistatic interactions between the MHC region and other genomic regions in hybrids or by negative pleiotropic effects of MHC haplotypes. Such pleiotropic effects appear to be common within species, as evidenced by multiple associations between the genomic MHC region and disease, including autoimmune disorders in humans (Dendrou et al. 2018). The analysis of genomic clines within contact zones suggests that the selective advantage of introgressing variants may indeed be substantial, aligning with previous findings in mice (Baird et al. 2012). This is because an increased rate of MHC introgression is observed, even though recombination within the zone typically does not operate long enough to free MHC variants from the genetic background involved in negative epistatic interactions.

The extensive variation in the number of MHC alleles per individual indicates that the investigated taxa differ in the complexity of the genomic MHC region. How might such heterogeneity in genetic architecture affect our results? The notable consistency among the three approaches, each of which was able to use only a subset of the taxa, indicates the overall robustness of our results, suggesting that increased introgression is a general feature of the MHC, regardless of its genomic complexity. An unknown, but in some taxa substantial, portion of the variation in MHC complexity may result from differences in the number of neutrally evolving pseudogenes and nonclassical MHC genes, which are likely evolving under purifying selection—our molecular methods did not allow unambiguous identification of classical MHC sequences. The most likely effect of this limitation would simply be a reduced power to detect adaptive introgression, making our approach conservative. In most jawed vertebrates, both MHC classes are tightly linked, making an independent assessment of their introgression challenging. In this context, results from *Solea*, a representative of teleost fishes that have unlinked MHC-I and MHC-II (Sato et al. 2000), are particularly informative. In this genus, both MHC classes show similar patterns suggesting they are subject to similar mechanisms promoting introgression.

Our work identifies introgression as a general mechanism by which species may acquire novel—and possibly also regain previously lost—MHC variants that may facilitate fighting pathogen assault and boost adaptive potential. We did not find evidence that the time since divergence between species substantially influences the relative strength of MHC introgression, indicating that adaptive introgression can persist for prolonged periods following the initial divergence. The asymmetry detected in the analysis of geographic clines suggests that the selective advantage of introgressed variants may be larger in less MHC-variable species. This pattern may indicate that demographic fluctuations or pathogen-mediated selection can often reduce MHC variation below optimal levels. Of course, hybridization may also result in the transfer of pathogens, leading to an interesting dynamic feedback between disease and introgression, as suggested in the case of human–Neanderthal hybridization (Enard and Petrov 2018; Greenbaum et al. 2019). The relationships between pathogen transmission and hybridization are, however, complex and still incompletely understood (Baird and Goüy de Bellocq 2019; Theodosopoulos et al. 2019). Despite this controversy, these findings have important implications for practical conservation, supporting the view that translocations and artificial or assisted introgression may be effective conservation measures in the age of the global biodiversity crisis (Hamilton and Miller 2016). This is especially relevant as the risks related to outbreeding depression and the general negative fitness effects of hybridization may be exaggerated (Aitken and Whitlock 2013; Ralls et al. 2018).

Widespread MHC introgression may contribute to the phenomenon of trans-species polymorphism—the long-term retention of allelic lineages through multiple speciation events, often observed in MHC and other genes evolving under long-term balancing selection (Radwan et al. 2020). The maintenance of trans-species polymorphisms by balancing selection alone appears problematic given the evidence for rapid turnover of MHC allelic lineages (Ejsmond et al. 2018; Herdegen-Radwan et al. 2021), but introgression can reintroduce lost lineages and lead to sharing of identical alleles. Finally, our results are relevant for understanding and predicting genomic patterns of divergence and gene flow in advanced stages of speciation, a topic that has been extensively studied in recent years (Butlin and Smadja 2018; Barton 2020; Kulmuni et al. 2020). According to the evidence accumulated so far, divergence along the genome in the late stages of speciation is generally high, indicative of multiple strong and coupled barriers to introgression that result in genome congealing (Barton 1983; Nosil et al. 2017). This landscape is occasionally dotted with small regions of increased introgression, which appear hybrid zone specific even in cases of parallel adaptive speciation (Ravinet et al. 2018). Recent progress in genomics should enable the inclusion of the MHC region in such scans of genomic divergence. If, as our results suggest, the MHC would be among the last genomic regions to stop introgressing between diverging taxa before reproductive isolation is complete, then it should repeatedly emerge as an outlier in surveys of divergence and gene flow in advanced stages of speciation.

Two potential limitations of our approach need to be considered. First, the coding of MHC genotypes as allele presence/absence and the subsequent analyses of the binary matrices could lead to inaccurate estimation of the hybrid index and to an erroneous inference of more extensive MHC introgression. The effect of binary coding of multilocus MHC genotypes was addressed by simulations, which showed that the hybrid index we calculated accurately reflected individual and population MHC ancestry, resulting in the correct estimation of cline parameters. In addition, randomization tests of MHC allele sharing did not use the MHC hybrid index and were therefore unaffected by potential issues related to its estimation. Second, it is in principle possible that more extensive MHC introgression, even if correctly inferred, is not due to the adaptive advantage of the introgressed alleles. The observed signal of more extensive MHC introgression could be a result of a parallel increase in the frequency of the same alleles, maintained as ancestral polymorphisms in both species, in the vicinity of the contact, possibly due to shared pathogen pressure. However, this scenario would require not only shared selection pressure but also the maintenance of a shared pool of identical alleles that can be selected in parallel in both species, which is unlikely (Dudek et al. 2019). Thus, considering the weight of the overall evidence, we conclude that processes other than adaptive MHC introgression are unlikely to explain the patterns detected in our study.

Adaptive MHC introgression may actually be even more pronounced than our results suggest. First, all of our approaches relied on the geographical structuring of introgressed variants, assuming that they were overrepresented in the vicinity of the contact. This implies ongoing or recent introgression that has not yet spread across the entire species ranges, as such spread would make past introgression difficult or impossible to detect. In such cases, the signal of historical introgression can still be detected by comparing MHC allele sharing between species with adjacent and nonadjacent ranges. Indeed, comparative analyses in *Triturus* newts (Gaczorek et al. 2023b) and *Podarcis* lizards (Gaczorek et al. 2023a) showed increased MHC allele sharing between species with adjacent ranges, even when considering only core range areas, unlikely to have been affected by recent introgression. The second indication that our approach may have underestimated MHC introgression comes from the *Lissotriton montandoni* × *L. vulgaris* hybrid zone, where multiple transects have been examined in detail (Dudek et al. 2019). In this system, interspecific MHC similarity at local scales exceeded similarity between geographic regions within species, indicating massive MHC introgression. At the same time, there was no difference in the width of MHC and genome-wide clines in any transect (Dudek et al. 2019). Reanalysis of the *Lissotriton* system presented here, using longer transects and more distant allopatric populations, provided some support for wider geographic MHC clines. If MHC introgressed extensively across the entire length of a transect, the actual wide geographic clines could be impossible to capture, as might be the case for two *Natrix* transects where the no-cline model fitted the observed MHC data best. However, in such cases, as previously suggested (Dudek et al. 2019), it may be possible to detect a shallow MHC cline forming de novo in the center of the hybrid zone due to restricted hybridization. This newly formed MHC cline may coincide with the genome-wide cline, giving a false impression of no increased MHC introgression.

A more nuanced and detailed picture of MHC introgression is likely to emerge in the future with the adoption of recent developments in long-read genomics (van Dijk et al. 2023) and associated analytical approaches (Eizenga et al. 2020; Dilthey 2021; Liao et al. 2023), which will provide direct access to long-range haplotype information (Ebert et al. 2021; Li et al. 2023). With the application of these methods at the population scales (De Coster et al. 2021), we should be able to directly trace the introgression of individual MHC haplotypes and their fragments, as they are progressively broken down by recombination. With such data, it will be possible to identify which haplotypes are introgressing adaptively and whether selection also prevents the introgression of some MHC haplotypes. The availability of population-level haplotype data will also allow us to study the effect of the genomic architecture of the MHC region, such as the number of genes, the size of the region, and the strength of linkage between MHC classes on introgression. While these advances will extend our understanding of introgression in general and MHC introgression in particular, they are unlikely to alter the overall conclusion of widespread adaptive MHC introgression.

In conclusion, we have found evidence supporting widespread adaptive introgression of MHC genes in numerous vertebrate hybrid zones. This finding establishes a much-needed generalization about the process of adaptive introgression, linking its many instances to a reasonably well-understood mechanism of rare allele advantage. We hypothesize that adaptive introgression is likely to be common in other genetic systems where balancing selection operates through rare allele advantage. Such genomic regions are expected to be among the last where gene flow persists during advanced stages of speciation. Our results indicate that adaptive MHC introgression is a major source of new adaptive MHC variation in natural systems, implying that assisted MHC introgression may be a valuable tool in practical conservation and management efforts.

## Materials and Methods

### Study Systems

We studied 29 hybrid zones (in cases where multiple geographically distant contacts between a pair of species were studied, they were considered separate zones) formed by 31 species representing 11 genera from major groups of jawed vertebrates: *Salmo* and *Solea* (teleost fishes); *Bombina* (anuran amphibians); *Lissotriton* and *Triturus* (urodele amphibians); *Emys* (turtles); *Anguis*, *Natrix*, and *Podarcis* (squamate reptiles); *Sphyrapicus* (birds); and *Myodes* (mammals) (supplementary table S1, Supplementary Material online). MHC introgression has been previously studied in three of the genera analyzed here: *Lissotriton* (Dudek et al. 2019), *Podarcis* (Gaczorek et al. 2023a), and *Triturus* (Gaczorek et al. 2023b). We combined published data with those from the eight genera obtained in this study and additional newly generated data from *Lissotriton*.

The time-calibrated phylogeny of the taxa included in this study is shown in Fig. 1a. This tree was used to control for the effect of phylogenetic relationships in the comparative analyses described below. The backbone phylogeny is from Irisarri et al. (2017), and divergence times within some major lineages and within genera were taken from the literature: Teleostei (Rabosky et al. 2018), Urodela (Palomar et al. 2021), *Bombina* (Pabijan et al. 2013), *Natrix* (Schöneberg et al. 2023), *Podarcis* (Yang et al. 2021), and *Triturus* (Wielstra et al. 2019). For all other genera not included in Irisarri et al. (2017), we obtained divergence times between closely related species from TimeTree (Kumar et al. 2017).

### Genome-Wide Admixture

For the majority of taxa, we used published SNP or microsatellite data (for references, see supplementary table S1, Supplementary Material online) that allowed an adequate estimation of the genome-wide interspecific admixture. For *Anguis* and *Clethrionomys*, admixture was estimated from SNP data for the purposes of this study (see the Supplementary Materials and Marková et al. 2020, 2023). Admixture estimates were expressed as *Q*-scores calculated using STRUCTURE (Pritchard et al. 2000), ADMIXTURE (Alexander et al. 2009), or NGSadmix (Skotte et al. 2013). For a part of *Sphyrapicus* samples, we used the genetic hybrid index estimates from Seneviratne et al. (2016), based on 180 SNPs, which we consider fully comparable to *Q*-scores. References, a summary of the markers and the proportion of admixed individuals, are given in supplementary table S1, Supplementary Material online.

To compare all hybrid zones under a similar and conservative regime, we treated all individuals with genome-wide admixture > 3% as admixed. This admixture threshold is often more stringent than the thresholds used in the original studies, which were tailored to the characteristics of the systems and the specific aims of the studies. Despite our best efforts, the sets of individuals genotyped for MHC and those with genome-wide admixture estimates did not always overlap completely, although overall genome-wide and MHC introgression were mostly estimated for the same individuals. In cases where genome-wide estimates were unavailable (e.g. *Salmo*), we treated an individual as nonadmixed if its population of origin was outside any contact zone or if the locality from which it originated contained only nonadmixed individuals of a single species. These cases are marked as “inferred” in supplementary table S5, Supplementary Material online. In addition, since the original studies often examined single hybrid zones involving only two species, we set the level of admixture from all other species to 0 for multispecies systems. We consider this approach justified because hybrid zones between different species in a genus are either geographically isolated (e.g. *Natrix*) or the original studies sampled transects beyond the areas of potential introgression with other species (e.g. *Sphyrapicus*). The compiled estimates of genome-wide admixture are given in supplementary table S5, Supplementary Material online.

### MHC Data

#### Samples

To genotype the MHC genes, we used previously obtained DNA samples for all taxa analyzed. We amplified the variable second exons of the MHC-I and MHC-IIB genes. The only exception was *Clethrionomys*, for which, due to primer availability, we amplified the third MHC-I exon and the second exon of one of the MHC-IIB genes (*DQB*). We aimed to maximize the number of populations while sampling several individuals from each locality to account for intrapopulation variation. The number of species, localities, and individuals for each taxon is shown in supplementary table S1, Supplementary Material online. Maps of sampled localities are provided in supplementary figs. S3 to S11, Supplementary Material online.

#### MHC Primers

The MHC data for *Lissotriton*, *Triturus*, and *Podarcis* were reported previously (Dudek et al. 2019; Gaczorek et al. 2023a, 2023b). For all other systems, including several additional allopatric *Lissotriton* populations located outside of previously analyzed transects, MHC genes were genotyped for the purpose of this study. Except for *Clethrionomys DQB* (Migalska et al. 2022), we used newly designed primers.

To design primers, we started by collecting available MHC sequences from as many species within a genus as possible to capture sequence variation in primer binding sites. For *Salmo*, this was straightforward as the sequences of *Salmo salar* MHC alleles used for primer design were retrieved from the IPD-MHC database (Maccari et al. 2017). For *Clethrionomys*, we used previously reported sequences of the third MHC-I exon (Migalska et al. 2017, 2019). In all other cases, we relied on existing genome or transcriptome assemblies (Benzekri et al. 2014; Nürnberger et al. 2021) or generated de novo transcriptome assemblies ourselves with Trinity (Grabherr et al. 2011) using publicly available RNAseq data. Due to the lack of genomic resources for *Anguis*, we sequenced transcriptomes of two *Anguis colchica* and two *Anguis fragilis* individuals (NCBI BioProject PRJNA1054985). With the assemblies in hand, we retrieved the MHC-I and MHC-IIB genes using tblastn or blastn from BLAST+ v2.9.0 (Camacho et al. 2009). As queries, we used sequences retrieved from Ensembl (Cunningham et al. 2022): human HLA-B (ENSG00000234745) and HLA-DRB1 (ENSG00000196126) proteins (*Solea*, *Bombina*, and *Anguis*), or sequences of the second exons of the MHC genes from *Chrysemys picta* (ENSCPBG00000005211, *Emys*) and *Gallus gallus* (ENSGALG00000033932, *Sphyrapicus*). For *Natrix*, we used published sequences of the second MHC exon from *Podarcis* (Gaczorek et al. 2023a).

In the next step, to increase the sensitivity of our searches, we extracted the second exons from the MHC genes identified in the previous step and used them as queries for an additional examination of focal assemblies (blastn). Finally, we designed primers in regions of low variation. For *Clethrionomys* MHC-I, to achieve satisfactory amplification, we also sequenced flanking introns of the third exon and designed primers overlapping them. In all other cases, the primers were located within the second exon. The sequences of primers, expected product lengths, and accession numbers for the raw sequencing data are given in supplementary table S6, Supplementary Material online.

#### MHC Genotyping

MHC exons were amplified in 10-μl PCR reactions containing: 50- to 100-ng genomic DNA, 5 μl of Multiplex PCR Kit (Qiagen) and primers at concentrations of 0.5 to 1 μM. Individuals were barcoded with a combination of 6-bp indexes at the 5′ end of the forward and reverse primers. PCR conditions for MHC-I amplification were as follows: initial denaturation at 95 °C for 15 min, followed by 33 cycles: 95 °C for 30 s, 56 °C for 30 s, and 72 °C for 70 s and final elongation at 72 °C for 10 min. PCR conditions for MHC-II amplification were as follows: initial denaturation at 95 °C for 15 min, followed by 35 cycles: 95 °C for 30 s, 55 °C for 30 s, and 72 °C for 70 s and final elongation at 72 °C for 10 min. Amplicons were pooled approximately equimolar based on gel-band intensity, pools were gel purified, and Illumina adapters were ligated using the NEBNext Ultra II DNA Library Prep Kit for Illumina (New England Biolabs) according to the manufacturer's protocol optimized for PCR-free workflow. Libraries were sequenced on an Illumina MiSeq using v2 500 cycle kits.

Genotyping was performed using the adjustable clustering method in AmpliSAS (Sebastian et al. 2016). Clustering and filtering parameters are detailed in supplementary table S2, Supplementary Material online. For *Emys*, we increased the *min_dominant_frequency_threshold* to 50% due to the overrepresentation of MHC clusters differing by a single base pair, indicating an elevated rate of sequencing errors. In the next step, we filtered out putatively nonfunctional alleles that differed by more than 6 bp from the expected product length or contained STOP codons or frameshift mutations. For each taxon and MHC class, we also adjusted the minimum per-amplicon frequency threshold to account for sequencing errors (supplementary table S2, Supplementary Material online) and filtered out all sequences that fell below this threshold. The threshold value was related to per-individual MHC diversity, with a higher threshold for taxa exhibiting lower diversity, indicating a lower number of MHC gene copies. Genotyping repeatability was estimated for multiple individuals (4 to 15) amplified and sequenced in two replicates by dividing the number of alleles detected in both replicates by the total number of alleles identified. Finally, the data were transformed into a binary presence–absence matrix, with the number of rows equal to the number of individuals and the number of columns equal to the number of alleles.

We omitted additional filtering steps applied elsewhere (Fijarczyk et al. 2018; Dudek et al. 2019; Gaczorek et al. 2023a, 2023b) that aimed to enrich the datasets for functional alleles of classical MHC genes by filtering out putatively nonclassical/nonfunctional variants based on expression information and phylogenetic clustering. Although these approaches are useful, they are highly taxon specific and their consistent application across a multitaxon data set was not feasible. Therefore, for *Lissotriton*, *Triturus* MHC-I, and *Podarcis*, for which MHC sequences have already been published, we included in our analyses all sequences categorized as putatively nonfunctional, except those containing STOP codons or frameshift mutations. For *Triturus* MHC-II, due to strong evidence for a single functional locus (Babik et al. 2009; Gaczorek et al. 2023b), we retained only MHC alleles assigned to this locus. The procedures described above used analytical settings tailored to the MHC characteristics of each taxon to achieve a single overarching goal—complete information about the MHC variants possessed by each individual. In other words, the heterogeneity of genotyping settings was necessary to achieve uniformity of MHC genotyping.

### Tests of MHC Introgression

To test for adaptive introgression of MHC genes, we applied three complementary analyses: MHC allele sharing, geographic clines, and genomic clines. However, due to the requirements of each approach, sample availability, and hybrid zone characteristics, we were not able to apply every test to each taxon. The list of taxa used for each test is shown in supplementary table S4, Supplementary Material online.

#### MHC Allele Sharing

If MHC introgression is adaptive, it should extend beyond areas of detectable genome-wide introgression. At the same time, the signal of ongoing or recent introgression should be stronger in the vicinity of the contact. Therefore, we expect to observe an excess of interspecific MHC allele sharing in the vicinity of the contact zone but beyond the actual hybrid zone, defined as the area of detectable genome-wide introgression (near) compared to areas further from the contact (far).

We only considered nonadmixed individuals from localities with an average admixture of <5%. Localities were classified as “near” or “far” based on their distance from the center of the hybrid zone, aiming for comparable sample sizes in both categories while ensuring that “far” populations were located away from areas of current and historical contact. A detailed description of the categorization for each taxon can be found in Supplementary Materials and figs. S3 to S11, Supplementary Material online. The significance of differences in allele sharing between species in “near” and “far” populations was tested with a permutation test, randomizing individuals within species between “near” and “far” populations 1,000 times.

For each hybrid zone and MHC class, we calculated a SES of differences in MHC allele sharing between “near” and “far” populations (Gotelli and McCabe 2002; Botta-Dukát 2018), which controls for variation in the permutation runs and, indirectly, for MHC variation in the system analyzed. This SES is a special case of Cohen's *d* effect size (Cohen 1988) where the size of one group (observed) is equal to 1. The absolute value of the SES measures the strength of the signal, while its sign indicates increased MHC allele sharing in “near” (+) or “far” populations (−).

#### Estimation of MHC Hybrid Index

For each MHC class and hybrid zone, we calculated the hybrid index (HI), which estimates the proportion of an individual's ancestry derived from one of the species, ranging between 0 and 1. The HI was estimated using the method suitable for dominant markers (Buerkle 2005). To obtain the reference allele frequencies for each species, we used all individuals from localities classified as “far.” For *Lissotriton*, reference allele frequencies were calculated separately for two zones (IN and OUT zones of Zieliński et al. 2019). The effect of binary coding of MHC genotypes on HI estimation was assessed with simulations (see below).

#### Geographic Clines

We designated transects, or pseudotransects in the case of *Triturus*, in which sampling deviated considerably from a 1D transect (Gaczorek et al. 2023b), through the hybrid zones (supplementary figs. S3 to S11, Supplementary Material online) and fitted clines to population means of (i) genome-wide admixture and (ii) the MHC HI, for each MHC class separately. Three models of smooth clines, i.e. without separate modeling the tails of the cline, were fitted with hzar (Derryberry et al. 2014): (i) ancestry fixed at 0 and 1 at the ends of the transect (”none”), (ii) estimated equal admixture at both ends of the transect (“fixed”), and (iii) admixture estimated separately at both ends of the transect (“free”). The best model was selected using the Akaike information criterion. We avoided using the single “free” model in all cases to prevent overfitting.

To summarize the difference in width and center between geographic MHC and genome-wide clines across multiple hybrid zones with a scale-free measure, we calculated Hedges *g* effect sizes (Hedges 1981; Rosenberg et al. 2013) for each MHC class in each hybrid zone. We conservatively assumed that the 2 log-likelihood intervals of the estimated cline parameters corresponded to 95% confidence intervals and used these to obtain the standard deviations necessary to calculate Hedges *g*. To test for the effect of MHC variation on cline shift, the difference between the centers of MHC and genome-wide clines was calculated such that its positive values indicated a shift of the cline center toward the less MHC diverse species.

#### The Effect of Binary Coding of Multilocus MHC Genotypes

Individual-based simulations were performed in SLiM 4 (Haller and Messer 2023). The ancestral population of *N* = 500 diploid individuals evolved for 20*N* = 10,000 generations before splitting into two descendant species (each with *N* = 500) that evolved in isolation. The MHC region contained five completely linked loci, each evolving according to the infinite alleles model with either (i) identical (θ_1-5_ = 0.6) or (ii) different (θ_1_ = 0.2, θ_2_ = 0.4, θ_3_ = 0.6, θ_4_ = 0.8, and θ_5_ = 1.0) population mutation rates under strong (*s* = 0.5) negative frequency dependence. Fitness of an allele was defined as *w* = 1 + *s*/2 − *fs*, where *f* is the allele frequency, giving the range of possible fitness values (1 − *s*/2; 1 + *s*/2). Each scenario was simulated 20 times. Transects through a clinal hybrid zone were constructed at three time points: 0.5, 1, and 4N generations after the split. Note that we were not attempting to simulate the formation and evolution of a cline but were interested in the effect of binary coding of MHC genotypes on the estimation of parameters of a known cline. At each time point, we created populations with expected admixture determined by position along the smooth cline (“none” model in hzar) of width *w* = 6 km, with the center in the middle of the transect. Fifteen equally spaced populations were formed within the 6-km center of the zone and ten equally spaced populations were formed at each 97-km tail of the transect. Each population contained ten individuals, composed of MHC haplotypes randomly sampled from the parental species, with the probability of a haplotype coming from each species determined by the locality's expected admixture proportion. The origin of each haplotype was known without error, so the actual admixture level of an individual and locality was calculated from these data and taken to represent genome-wide admixture (“known-ancestry”). The HI for each individual was calculated from its complete MHC genotype, i.e. considering each locus separately and assigning alleles to their loci of origin (“MHC genotype”), as well as multilocus MHC genotypes binary coded as described above (“MHC binary”). Reference allele frequencies were estimated from 30 individuals randomly sampled from each parental species. Thus, some alleles present in individuals in the transect had unknown reference frequencies and were ignored in the calculation of HI, as was the case in the analysis of empirical data.

#### Genomic Clines

To examine MHC ancestry relative to genome-wide ancestry in admixed individuals, we used a genomic cline approach—Barton's concordance analysis (Szymura and Barton 1986). For each hybrid zone and MHC class, we fitted, using maximum likelihood in R v. 4.2.2, the equation modeling individual MHC ancestry as a function of genome-wide ancestry: $p_{\mathrm{MHC}}=\bar{\;p}+2\bar{\;p}\bar{q}(\alpha +(\bar{\;p}-\bar{q})\beta )$ (Szymura and Barton 1986), where $p_{\mathrm{MHC}}$ is the proportion of the individual's MHC ancestry derived from species 1, $\bar{\;p}$ is the proportion of genome-wide ancestry derived from species 1, $\bar{q}$ = $\left(1-\bar{\;p}\right)$ is the proportion of genome-wide ancestry derived from species 2, and *α* and *β* are the genomic cline parameters. The *α* parameter measures a relative excess of MHC ancestry from one of the two species (genomic cline “shift”); in our case, a positive *α* always indicated an excess of ancestry from the more MHC diverse species. The *β* parameter measures the relative rate of transition in ancestry (genomic cline “width”), with *β* < 0 indicating a slower rate of transition in MHC, i.e. more MHC admixture than expected from genome-wide admixture. The genomic cline curve is characterized by considerable flexibility and fixed ends at points (0, 0) and (1, 1), corresponding to individuals without genome-wide admixture, which do not contribute to the cline fitting in this model. Note that we could not use the more recently developed approaches to fit genomic clines (Gompert and Buerkle 2011) as they require allele counts at individual loci, which were not available. The 2 log-likelihood parameter intervals were taken as a proxy for 95% confidence intervals, which we used to calculate the variances of the estimates. As the parameters of genomic clines are always measured on the same scale, the estimates and their variances were used directly in subsequent modeling.

#### Comparative Analysis

The significance of the signal of increased MHC introgression across taxa was tested using PGLS with the ape (Paradis and Schliep 2019), matrix (Bates et al. 2024), and nlme (Pinheiro et al. 2023) R packages. The time-calibrated phylogeny (Fig. 1) was used to obtain the expected correlation matrix between pairs of hybridizing species (note that pairs of species, not individual species, are the units of measurement here). The matrix of cophenetic distances, which summed the branch lengths between nodes representing the most common ancestors of hybridizing species, was transformed into the correlation matrix by dividing the distance between nodes by the cophenetic distance between the most distant species. The strength of the phylogenetic signal was estimated using Pagel's *λ*.

All models included SESs or genomic cline parameters as response variables and MHC class as an explanatory variable. Where parameters were estimated for multiple transects for the same species pair, both estimates and their variances were averaged. In a subset of models, we also included time of divergence between hybridizing species and the MHC class × time of divergence interaction. Models with and without interaction were compared using LRT in R anova(); and if interaction did not significantly improve fit, it was omitted. We assumed no phylogenetic correlation between MHC classes, and the effect of phylogeny within each class was included as described above. The model also included weights corresponding to the inverse of the variation in the estimates, accounting for their uncertainty. For randomization tests, the weights were not applied because a large and constant number of permutations consistently resulted in nearly identical variances (≈1) of SES for any reasonable value of effect size.

## Supplementary Material

msae201_Supplementary_Data

## Data Availability

The data underlying this article are available in the article, in its online Supplementary material, at: https://github.com/TomekGa/MHC_comparative_analysis and in NCBI BioProject PRJNA1054985. The code is at: https://github.com/TomekGa/MHC_comparative_analysis.
