# Supplementary material for: Widespread Adaptive Introgression of Major Histocompatibility Complex Genes across Vertebrate Hybrid Zones
Source: Mol Biol Evol. 2024 Sep 26;41(10):msae201. doi: 10.1093/molbev/msae201 (PMC11472244; doi:10.1093/molbev/msae201)
Supplement: msae201_Supplementary_Data [file msae201_supplementary_data.zip › Gaczorek_et_al_MHC_introgression_Supplementary_Materials_Rev1.pdf]

Supplementary Materials

to

Widespread adaptive introgression of MHC genes across vertebrate hybrid zones

Description of categorization of non-admixed populations into “near” and “far” categories.

Salmo – Although we lacked genome-wide estimates of admixture based on genetic markers, we can clearly distinguish nonadmixed, “far” populations because they occupy isolated rivers (*S. salar*) or parts of rivers above barriers, that are inaccessible to the other species (*S. trutta*, Fig. S3). However, we lack non-admixed “near” populations, as all the remaining genotyped individuals could have potentially been admixed. Therefore, we perform a randomisation test using sympatric rather than “near” individuals. This allowed to test for MHC introgression, but not for its adaptiveness.

Solea – The division between “near” and “far” is shown in Fig S4. The wide “near” is an effect of limited but still detectable admixture near Annaba, Algeria.

Bombina - The division between “near” and “far” is shown in Fig S5. Note that due to the limited number of non-admixed populations, we analysed Bochnia and Chernivitsi transects together[1].

Lissotriton - The “far” populations are shown in Fig S6 and are located outside of analysed transects. Note that the set of “far” *L. vulgaris* populations differed between eastern and western transects, because the two L. vulgaris lineages are strongly divergent as inferred by Zieliński [2,3] (IN and OUT zone).

Triturus – The “far” populations correspond to alloF populations from[4]. Note that we analysed only four Balkan hybrid zones as the level of genome-wide admixture for the Western European hybrid zones was estimated based on allozymes which we consider of insufficient resolution for comparative analysis.

Anguis – The division between “near” and “far” is shown in Fig S7.

Emys – The “far” populations are shown in Fig S8. Note that for the *E. orbicularis* – *E. trinacris* hybrid zone, we could not obtain individual Q-scores. Thus, we classified “far” populations

solely by geography, based on Figure 2 from[5]. Due to the lack of the "near" category, similar to *Salmo*, we compared "far" with sympatry and used the test results only if they were not significant.

Natrix - The "far" populations are shown in Fig S9.

Podarcis – The "far" populations correspond to allopatric populations in[6] .

Sphyrapicus - The "far" populations are shown in Fig S10.

Clethrionomys - The "far" populations are shown in Fig S11.

Genome-wide data newly generated for this study

Clethrionomys

Whole genome sequencing data were generated from 28 red-backed voles (*C. rutilus*) and 20 bank voles (*C. glareolus*) using the NEBNext Ultra II FS DNA Library Prep Kit (New England Laboratories, Woburn, MA) and 150 bp paired-end sequencing on the Illumina NovaSeq 6000 platform at the OMRF DNA Sequencing Facility, University of Oklahoma, USA (for details, see[7]). The trimmed reads were then mapped to the bank vole reference genome[7] using the bwa-mem algorithm from bwa (v. 0.7.10-r789) (Li et al. 2013). SNPs and genotype likelihood were called using angsd (v. 0.934)[8] with the following criteria (-GL 2 -doMajorMinor 1 -doMaf 1 -SNP_pval 2e-6 -minMapQ 30 -minQ 30 -minInd 48 -minMaf 0.05 -doGlf 2). The final dataset with 13,464,826 SNPs was used to estimate the admixture proportion in NGSadmix (v. 32)[9].

Anguis

Hybrid indices were estimated for *Anguis* individuals using 90 "species-diagnostic" SNPs assayed by LGC Biosearch Technologies using the Kompetitive Allele Specific PCR (KASP) protocol[10,11]. The 51bp KASP SNP probes were designed by SJEB to lie within the (~1800bp) sequences of genome-wide anchored hybrid enrichment loci that diagnosed *Anguis colchica* from *A. fragilis* samples[12]. SNP state associated with *A. fragilis* was verified with *diem*[13], and hybrid index calculated as the proportion of *A. fragilis* SNP states.

Supplementary Tables

Table S1. Sampling. No. hybrid zones – the number of contact zones studied in the genus; in some cases more than one contact between a pair of species was studied; Number of localities – the number of all localities with at last one individual of a given species; Number of ind. – number of individuals exhibiting > 50% ancestry from a given species; "inferred" in "Genome-wide markers" refers to individuals which were assumed to be non-admixed (see *Genome-wide admixture* section of *Methods*); References – the source of information about samples and genome-wide admixture estimates.

Genus	No. hybrid zones	Species	Number of localities	Number of ind.	Genome-wide markers (No. of ind.) *	% of admixed ind. **	Geographic region	References
*Salmo****	1	*salar*	10	96	inferred (93)	-	Island of Newfoundland, Canada	this study
		trutta	14	190			Ireland	
Solea	1	*aegyptiaca*	6	77	10,759 SNP (137)<br>inferred (9)	12.4	Northern and Eastern coast of Africa	[14]
		senegalensis	6	68				
Bombina	2	*bombina*	18	54	1883 SNP (56)<br>653 SNP (39)<br>inferred (56)	25.2	Carpathians	[1], this study
		variegata	28	97				
Lissotriton	4	*montandoni*	90	980	2849 SNP (1618)<br>>20,000 SNP (12)<br>inferred (568)	24.2		[2,15,16]
		vulgaris	123	1218				
Triturus	4	*anatolicus*	20	56	49 SNP (154)<br>52 SNP (149)<br>inferred (33)	28.9	Balkans and Anatolia	[17,18]
		cristatus	17	55				
		ivanbureschi	40	107				
		macedonicus	42	112				
Anguis	1	*colchica*	118	227	>= 80 SNP (264)<br>< 80 SNP (15)<br>inferred (29)	71.1	Czechia and Poland	this study
		fragilis	73	81				
Emys	2	*o. occidentalis*	24	136	15 microsatellites (239)<br>inferred (53)	3.8	Apennine Peninsula & Pyrenees	[19]
		*orbicularis*****	30	132				
		trinacris	7	24				
Natrix	4	*astreptophora*	13	15	13 microsatellites (270)	27.0	Europe	[20-24]

		helvetica	113	135				
		natrix	92	120				
Podarcis	6	*bocagei*	17	39	16739 SNP (61)<br>6905 SNP (67)<br>4266 SNP (18)<br>1341 SNP (42)<br>> 500 SNP (151)<br>Inferred (198)	2.8	Iberian Peninsula	[25-28]
		carbonelli	29	30				
		guadarramae	13	68				
		hispanicus	13	24				
		liolepis	35	126				
		vaucheri	12	80				
		virescens	42	170				
Sphyrapicus	3	*nuchalis*	92	115	180 SNP (155)<br>32136 (36)<br>Dozens SNP (114)<br>inferred (30)	70.5	Canada and USA	[29-32]
		ruber	117	146				
		varius	60	69				
Clethrionomys	1	*glareolus*	6	60	13.5 million SNP (32)<br>6078 SNP (30)<br>inferred (24)	1.2	Fennoscandia	[33], this study
		rutilus	3	26				

* The numbers in parentheses include only individuals successfully genotyped for MHC. In many systems, e.g. *Natrix*, genome-wide admixture estimates were available for more individuals. Those records were used solely for the analysis of geographic clines to increase resolution. All records used for this purpose can be found in Table S4.

** Admixture > 3%

*** We lack direct estimates for genome-wide admixture in *Salmo*. However, *S. salar* and *S. trutta* can be unambiguously distinguished based on morphology, while non-admixed populations can be identified based on the presence of impassable barriers within rivers (*S. trutta)* or the lack of allospecifics in isolated rivers (*S. salar)*.

**** It includes three subspecies of *E. orbicularis*, which are known to hybridize extensively (*E. o. galloitalica*, *E. o. hellenica*, *E. o. orbicularis)*.

Table S2 – Parameters of the Amplisas software used for MHC genotyping and estimated genotyping repeatability. Due to its large size the table is in a separate Excel file.

Table S3. MHC diversity. Allelic richness was calculated for the sample of 8 individuals.

Genus	Species	Number of MHC-I alleles		Number of MHC-II alleles		Mean number of MHC-I alleles per individual		Mean number of MHC-II alleles per individual		MHC-I allelic richness	MHC-II allelic richness
Salmo	*salar*	27	57	26	44	5.50	4.99	2.66	2.43	16.50	11.82
	trutta	30		18		4.24		2.00		16.32	8.66
Solea	*aegyptiaca*	119	172	74	108	6.15	5.98	3.19	3.15	28.79	18.11
	senegalensis	90		49		5.54		3.10		26.62	14.48
Bombina	*bombina*	71	127	26	46	13.78	14.87	5.06	4.74	40.71	15.56
	variegata	112		37		15.56		4.40		55.60	15.62
Lissotriton	*montandoni*	615	1059	171	331	13.04	13.08	3.47	3.58	75.64	20.93
	vulgaris	778		242		13.11		3.67		66.19	18.30
Triturus	*anatolicus*	281	813	28	60	18.09	18.05	1.88	1.81	80.89	10.13
	cristatus	229		20		19.06		1.88		93.94	10.12
	ivanbureschi	239		14		17.70		1.55		65.62	4.98
	macedonicus	190		6		17.87		2.00		67.99	4.36
Anguis	*colchica*	136	178	129	162	23.72	23.54	18.97	17.27	74.84	59.54
	fragilis	114		57		23.31		13.67		71.48	33.76
Emys	*o. occidentalis*	21	37	11	50	8.46	7.62	1.49	2.03	12.96	4.70
	orbicularis	27		42		6.40		2.38		12.32	12.31
	trinacris	17		12		7.77		2.21		14.68	7.26
Natrix	*astreptophora*	9	47	81	555	3.93	3.73	31.27	34.64	9.00	81.00
	helvetica	34		331		3.77		33.70		10.85	119.24
	natrix	35		401		3.67		35.74		11.08	142.55
Podarcis	*bocagei*	141	1827	104	1400	16.75	17.68	9.61	12.28	73.10	44.94
	carbonelli	182		80		19.67		9.71		102.18	44.97
	guadarramae	258		179		19.75		12.08		93.36	60.02
	hispanicus	168		105		18.11		12.40		90.96	57.02
	liolepis	600		379		17.34		14.75		108.64	85.79
	vaucheri	304		251		15.79		10.20		88.55	61.21
	virescens	711		481		17.66		11.60		109.78	65.70
Sphyrapicus	*nuchalis*	81	139	27	60	14.61	14.42	6.65	6.54	45.34	15.24
	ruber	91		30		14.58		6.31		48.92	16.12
	varius	80		37		13.68		6.76		52.44	23.14
Clethrionomys	*glareolus*	420	526	87	102	34.23	33.66	5.46	5.53	162.40	31.42
	rutilus	203		45		32.25		5.68		122.16	26.42

Table S4. Taxa used in different tests of adaptive MHC introgression. Whenever a given taxon was not used in a test, we provided a brief explanation.

Taxon	Randomization test	Geographic clines	Genomic clines
Salmo	The lack of non-admixed populations in the proximity of contact	Limited availability of samples	Limited number of individuals with genome-wide admixture
Solea	YES	YES	YES
Bombina	YES	YES	YES
Lissotriton	YES	YES	YES
Triturus	YES	YES	YES
Anguis	YES	YES	YES
Emys	YES	YES (only for *E. o. occidentalis* – *E. orbicularis* hybrid zone)	Limited number of individuals with genome-wide admixture
Natrix	YES	YES	YES
Podarcis	YES	Impossible to reduce spatial complexity to a one-dimensional transect	Limited number of individuals with genome-wide admixture
Sphyrapicus	YES	YES (only for *S. nuchalis* – *S. ruber* hybrid zone)	YES
Clethrionomys	YES	Limited availability of samples	Limited number of individuals with genome-wide admixture

Due to their large size Tables S2, S5 and S6 are in a separate Excel file

Table S2 – Parameters of the Amplisas software used for MHC genotyping and estimated genotyping repeatability.
Table S5 – Estimates of genome-wide admixture.
Table S6 – Primers used to amplify second (third in the case of *Clethrionomys* MHC-I) exons of MHC genes in all taxa analysed. The table also includes the source of the original sequences used for primer design and the expected length of the product.

Supplementary Materials References

1 Dufresnes, C. *et al.* Revisiting a speciation classic: comparative analyses support sharp but leaky transitions between *Bombina* toads. *J Biogeogr.* **48**, 548-560 (2021).

2 Zieliński, P. *et al.* Differential introgression across newt hybri zones - evidence from replicated transects. *Mol Ecol.* **28**, 4811-4824 (2019).

3 Zieliński, P., Nadachowska-Brzyska, K., Dudek, K. & Babik, W. Divergence history of the Carpathian and smooth newts modelled in space and time. *Mol Ecol.* **25**, 3912-3928 (2016).

4 Gaczorek, T. S. *et al.* Interspecific introgression of MHC genes in *Triturus* newts: Evidence from multiple contact zones. *Mol Ecol.* **32**, 867-880 (2023).

5 Vamberger, M. *et al.* Differences in gene flow in a twofold secondary contact zone of pond turtles in southern Italy (Testudines: Emydidae: *Emys orbicularis galloitalica*, *E. o. hellenica*, *E. trinacris*). *Zool Scr.* **44**, 233-249 (2015).

6 Gaczorek, T. S. *et al.* Widespread introgression of MHC genes in Iberian Podarcis lizards. *Mol Ecol.* **32**, 4003-4017 (2023).

7 Marková, S. *et al.* Local adaptation and future climate vulnerability in a wild rodent. *Nat Comm.* **14**, 7840 (2023).

8 Korneliussen, T. S., Albrechtsen, A. & Nielsen, R. ANGSD: analysis of next generation sequencing data. *BMC Bioinf.* **15**, 356 (2014).

9 Skotte, L., Korneliussen, T. S. & Albrechtsen, A. Estimating individual admixture proportions from next generation sequencing data. *Genetics* **195**, 693-702 (2013).

10 He, C., Holme, J. & Anthony, J. SNP genotyping: the KASP assay. In: Fleury, D. and R. Whitford (eds.), Crop Breeding: Methods and Protocols. *Meth Mol Biol.*, **1145**, 75-86 (2014).

11 Semagn, K., Babu, R., Hearne, S. & Olsen, M. Single nucleotide polymorphism genotyping using Kompetitive Allele Specific PCR (KASP): overview of the technology and its application in crop improvement. *Mol Breed.* **33**, 1-14 (2014).

12 Gvoždík, V. *et al.* Phylogenomics of *Anguis* and *Pseudopus* (Squamata, Anguidae) indicates Balkan-Apennine mitochondrial capture associated with the Messinian event. *Mol Phyl Evol.* **180**, 107674 (2023).

13 Baird, S. J., Petružela, J., Jaroň, I., Škrabánek, P. & Martínková, N. Genome polarisation for detecting barriers to geneflow. *Meth Ecol Evol.* **14**, 512-528 (2023).

14 Souissi, A., Bonhomme, F., Manchado, M., Bahri-Sfar, L. & Gagnaire, P.-A. Genomic and geographic footprints of differential introgression between two divergent fish species (*Solea* spp.). *Heredity.* **121**, 579-593 (2018).

15 Fijarczyk, A., Dudek, K., Niedzicka, M. & Babik, W. Balancing selection and introgression of newt immune-response genes. *Proc Roy Soc B.* **285**, 20180819 (2018).

16 Wielstra, B. *et al.* The distributions of the six species constituting the smooth newt species complex (*Lissotriton vulgaris* sensu lato and *L. montandoni*)–an addition to the New Atlas of Amphibians and Reptiles of Europe. *Amphibia-Reptilia* **39**, 252-259 (2018).

17 Wielstra, B., Burke, T., Butlin, R. & Arntzen, J. A signature of dynamic biogeography: enclaves indicate past species replacement. *Proc Roy Soc B.* **284**, 20172014 (2017).

18 Wielstra, B. *et al.* A genomic footprint of hybrid zone movement in crested newts. *Evol Lett.* **1**, 93-101 (2017).

19 Pöschel, J. *et al.* Complex hybridization patterns in European pond turtles (*Emys orbicularis*) in the Pyrenean Region. *Sci Rep.* **8**, 15925 (2018).

20 Asztalos, M. *et al.* It takes two to tango-Phylogeography, taxonomy and hybridization in grass snakes and dice snakes (Serpentes: Natricidae: *Natrix natrix*, *N. tessellata*). *Vert Zool.* **71**, 813-834 (2021).
21 Asztalos, M. *et al.* How often do they do it? An in-depth analysis of the hybrid zone of two grass snake species (*Natrix astreptophora* and *Natrix helvetica*). *Biol J Linn Soc.* **131**, 756-773 (2020).
22 Kindler, C. *et al.* Hybridization patterns in two contact zones of grass snakes reveal a new Central European snake species. *Sci Rep.* **7**, 7378 (2017).
23 Schultze, N., Laufer, H., Kindler, C. & Fritz, U. Distribution and hybridisation of barred and common grass snakes (*Natrix helvetica*, *N. natrix*) in Baden-Württemberg, South-western Germany. *Herpetozoa* **32**, 229-236 (2019).
24 Schultze, N. *et al.* Mitochondrial ghost lineages blur phylogeography and taxonomy of *Natrix helvetica* and *N. natrix* in Italy and Corsica. *Zool Scri.* **49**, 395-411 (2020).
25 Caeiro-Dias, G. *et al.* Variable levels of introgression between the endangered *Podarcis carbonelli* and highly divergent congeneric species. *Heredity* **126**, 463-476 (2021).
26 Caeiro-Dias, G., Brelsford, A., Meneses-Ribeiro, M., Crochet, P. A. & Pinho, C. Hybridization in late stages of speciation: Strong but incomplete genome-wide reproductive isolation and 'large Z-effect'in a moving hybrid zone. *Mol Ecol.* **32**, 4362-4380 (2023).
27 Caeiro-Dias, G. *et al.* Nuclear phylogenies and genomics of a contact zone establish the species rank of *Podarcis lusitanicus* (Squamata, Lacertidae). *Mol Phyl Evol.* **164**, 107270 (2021).
28 Kaliontzopoulou, A., Pinho, C., Harris, D. J. & Carretero, M. A. When cryptic diversity blurs the picture: a cautionary tale from Iberian and North African *Podarcis* wall lizards. *Biol J Linn Soc.* **103**, 779-800 (2011).
29 Natola, L., Billerman, S. M., Carling, M. D., Seneviratne, S. S. & Irwin, D. Geographic variability of hybridization between red-breasted and red-naped sapsuckers. *Evolution* **77**, 580-592 (2023).
30 Natola, L., Curtis, A., Hudon, J. & Burg, T. M. Introgression between *Sphyrapicus nuchalis* and *S. varius* sapsuckers in a hybrid zone in west-central Alberta. *J Avian Biol.* **52,** e02717 (2021).
31 Natola, L., Seneviratne, S. S. & Irwin, D. Population genomics of an emergent tri-species hybrid zone. *Mol Ecol.* **31**, 5356-5367 (2022).
32 Seneviratne, S. S., Davidson, P., Martin, K. & Irwin, D. E. Low levels of hybridization across two contact zones among three species of woodpeckers (*Sphyrapicus* sapsuckers). *J Avian Biol.* **47**, 887-898 (2016).
33 Marková, S. *et al.* High genomic diversity in the bank vole at the northern apex of a range expansion: The role of multiple colonizations and end-glacial refugia. *Mol Ecol.* **29**, 1730-1744 (2020).

Supplementary Figures

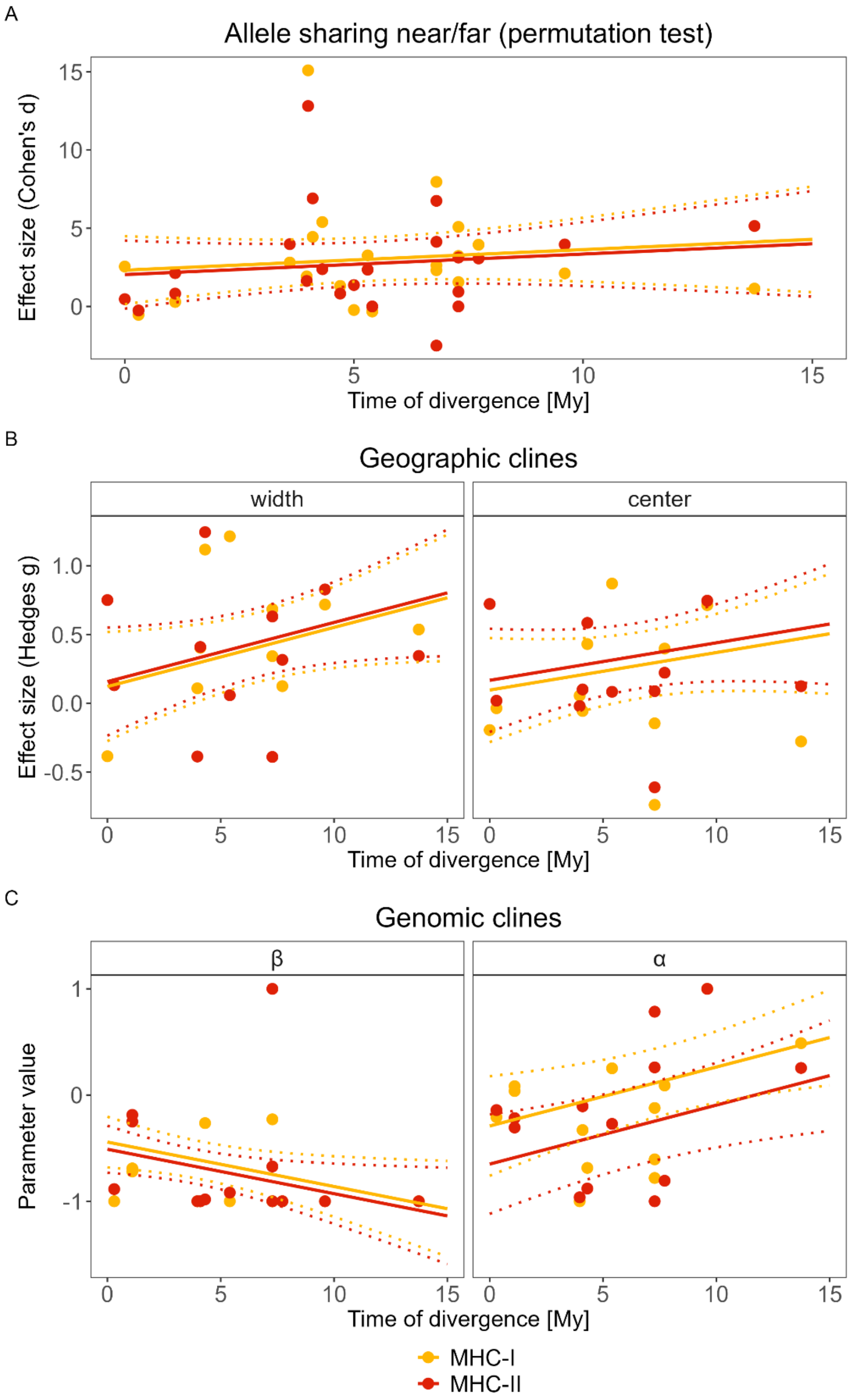

Figure S1. The effect of the time of divergence between hybridising species on the difference between MHC and genome-wide introgression. A) The difference in allele sharing near and far from the contact zone; B) Geographic clines; C) genomic clines. Lines show predictions from the model (in all cases models without interaction were selected by the likelihood-ratio test) and dotted lines represent 95% confidence intervals.

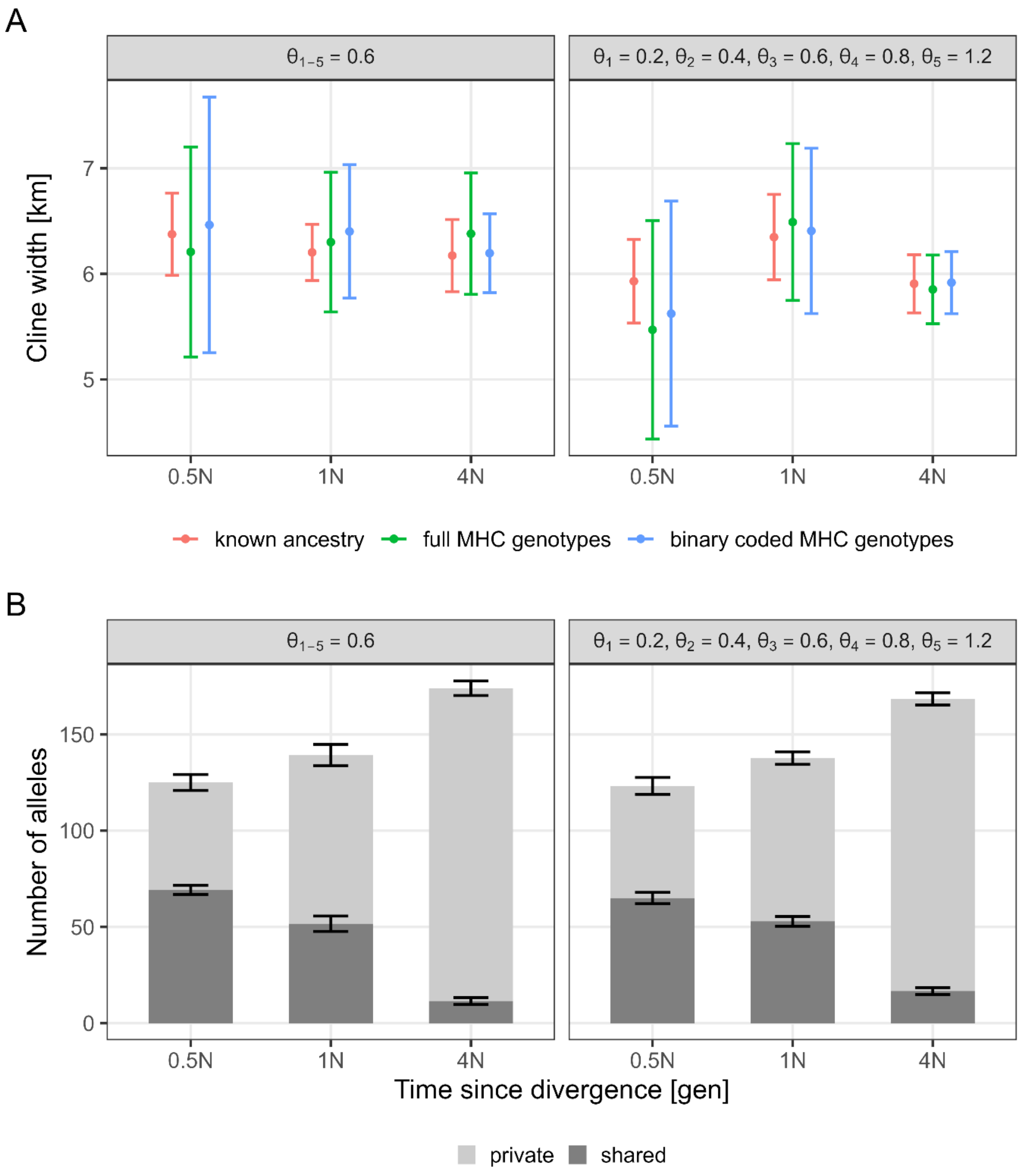

Figure S2. Results of simulations testing the effect of binary coding of multilocus MHC genotypes on the estimation of geographic cline parameters. Results are shown for hybrid zones formed after 0.5N, 1N and 4N generations of evolution in isolation. A) Estimated cline widths and associated 95% confidence intervals; θ - population mutation rate (4Neμ), left panel – equal mutation rates of all MHC loci, right panel – mutation rate differs between loci; known ancestry – species of origin of each MHC haplotype is known without error, full MHC genotypes – hybrid index estimated from known genotypes at each locus, binary coded MHC genotypes – hybrid index estimated considering each MHC allele as an independent binary dominant locus. B) Mean number of MHC alleles, private and shared between species, summed over the five loci, with associated 95% confidence intervals.

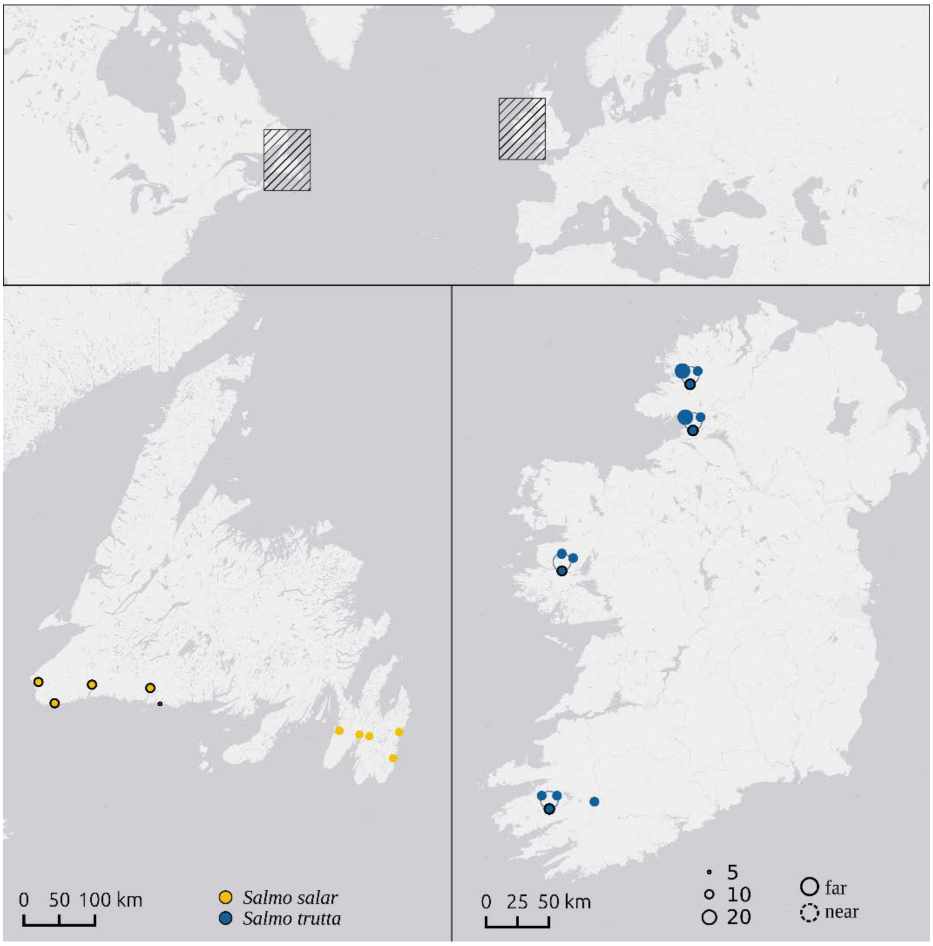

Figure S3 – *Salmo*. Sampling, genome-wide ancestry, and classification of populations into "near" and "far" .

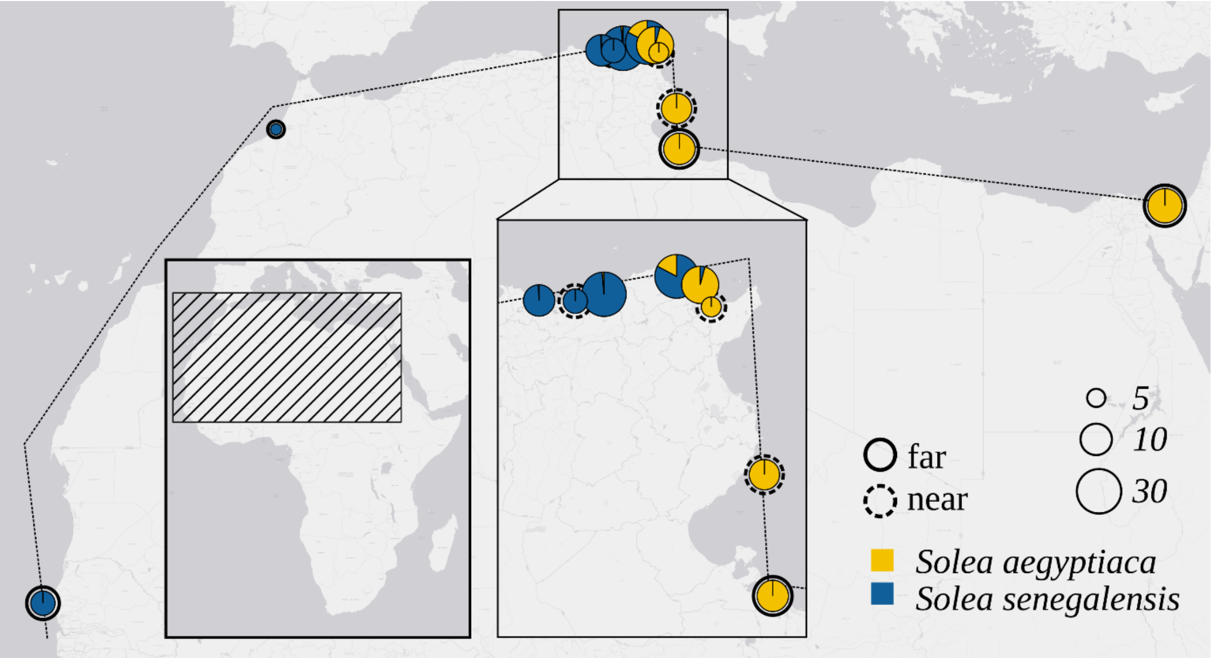

Figure S4 – *Solea*. Sampling, genome-wide ancestry, classification of populations into “near” and “far” , and designated transect.

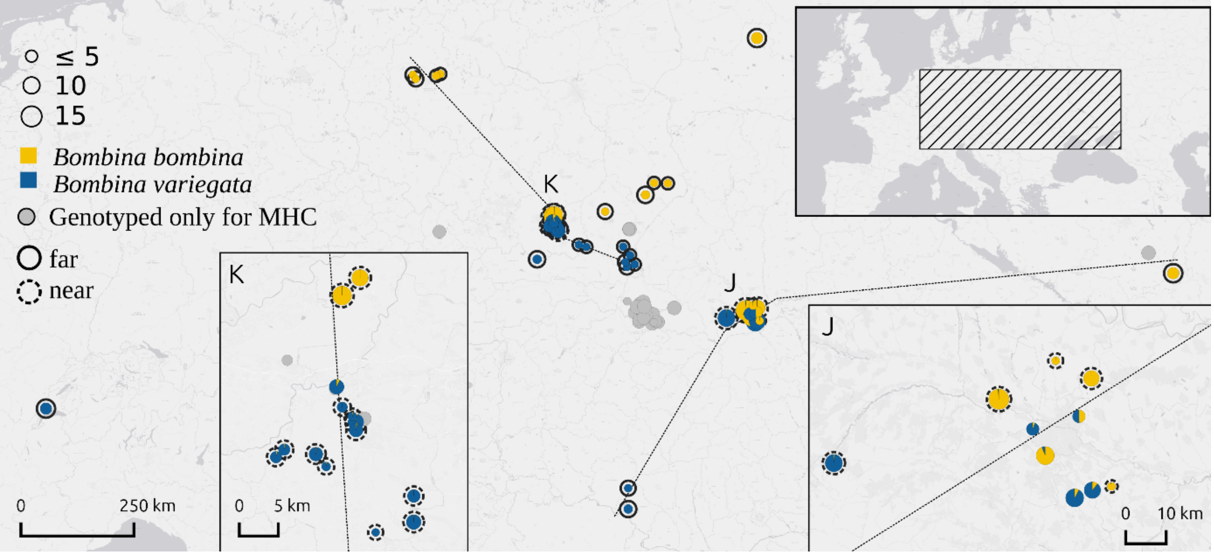

Figure S5 – *Bombina*. Sampling, genome-wide ancestry, classification of populations into "near" and "far" , and designated transects.

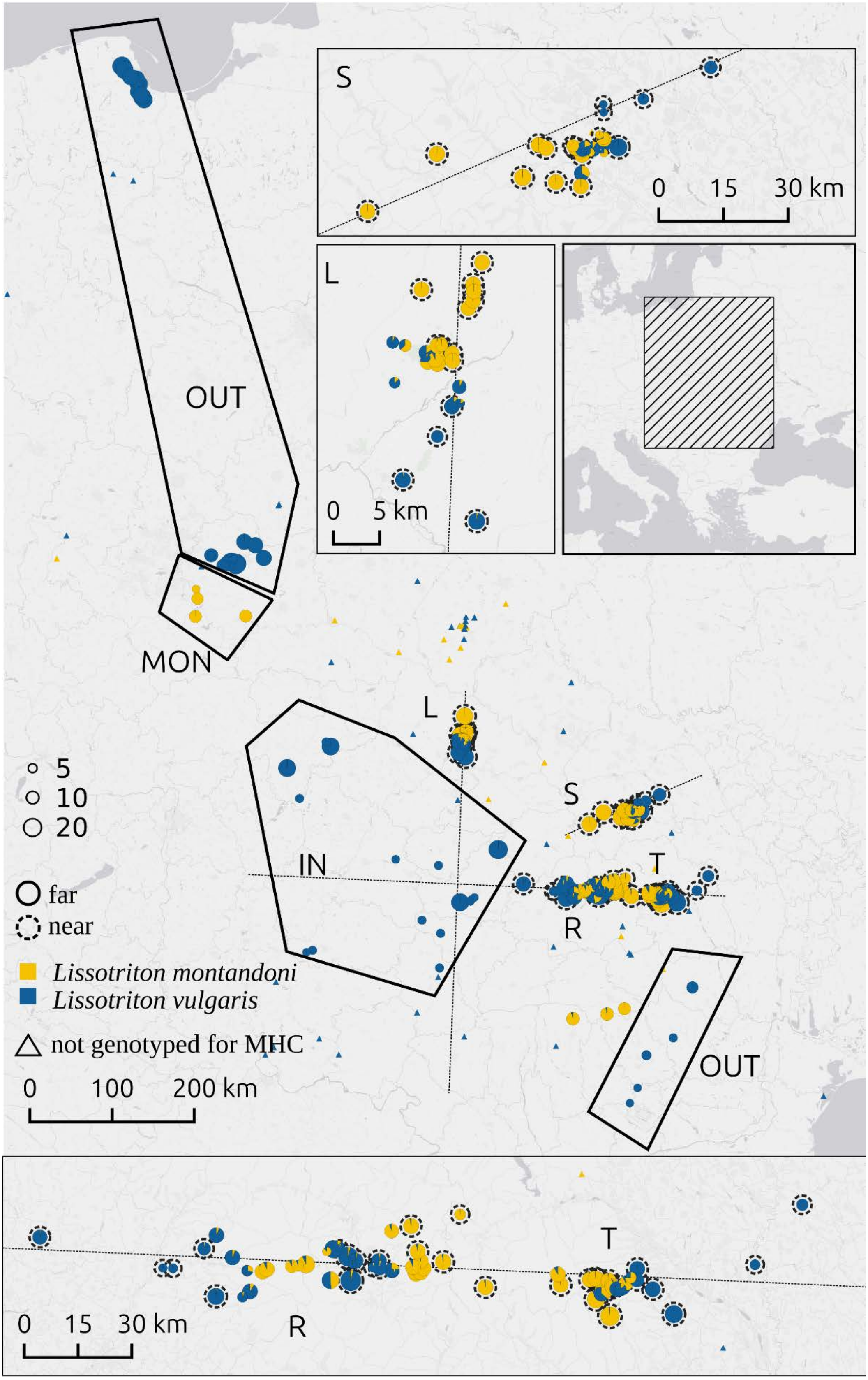

Figure S6 – *Lissotriton*. Sampling, genome-wide ancestry, classification of populations into "near" and "far" , and designated transects. Note distinct sets of "far" populations in IN and OUT zones.

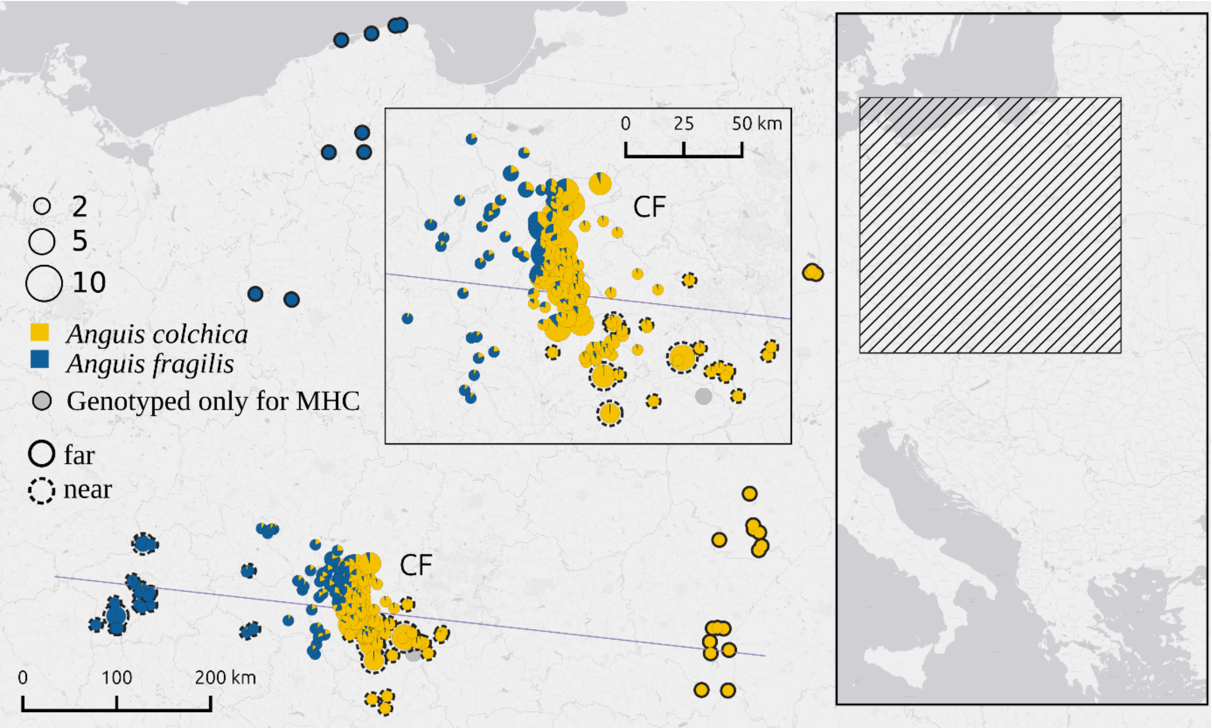

Figure S7 – *Anguis*. Sampling, genome-wide ancestry, classification of populations into "near" and "far" , and designated transect.

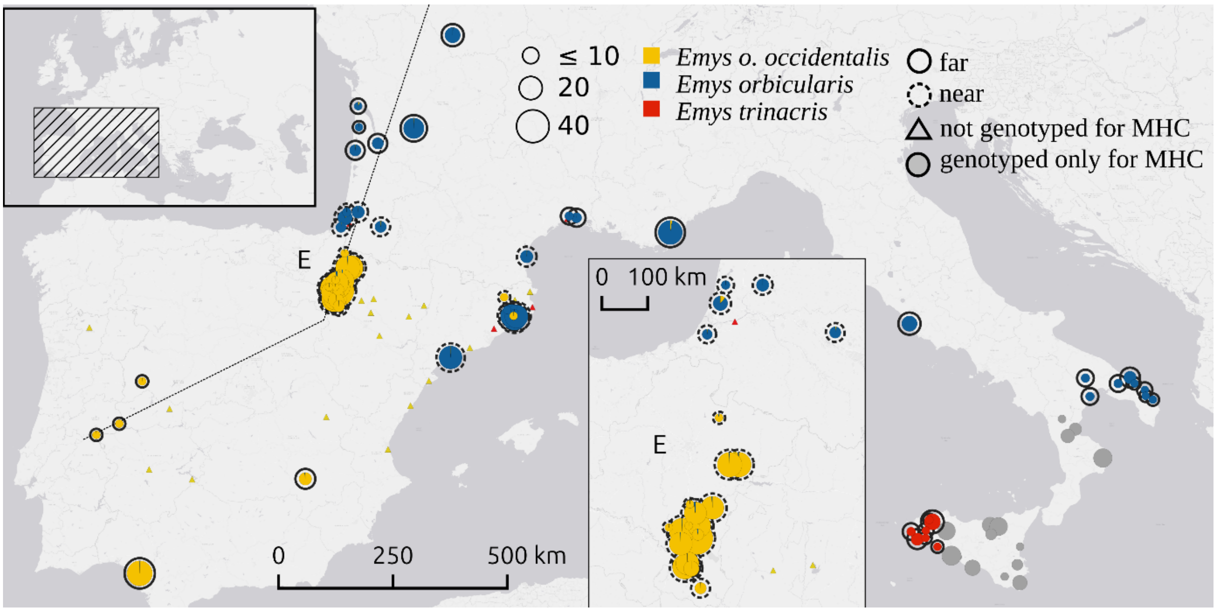

Figure S8 – *Emys*. Sampling, genome-wide ancestry, classification of populations into "near" and "far" , and designated transect.

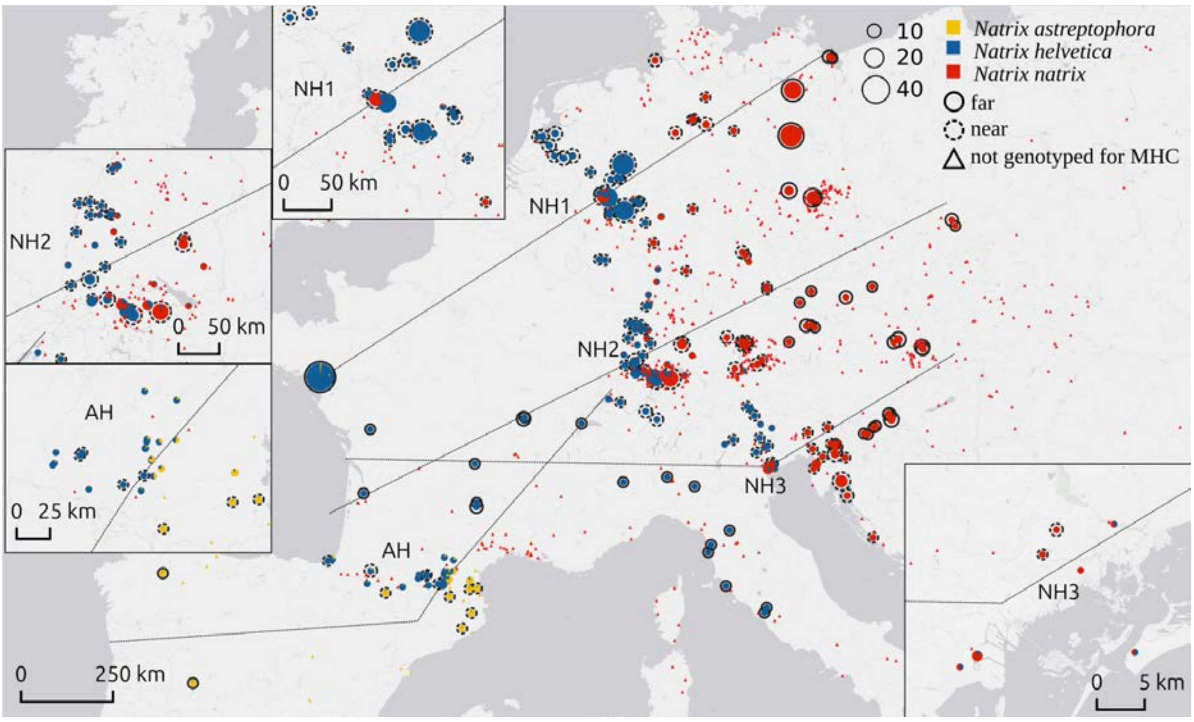

Figure S9 – *Natrix*. Sampling, genome-wide ancestry, classification of populations into "near" and "far" , and designated transects.

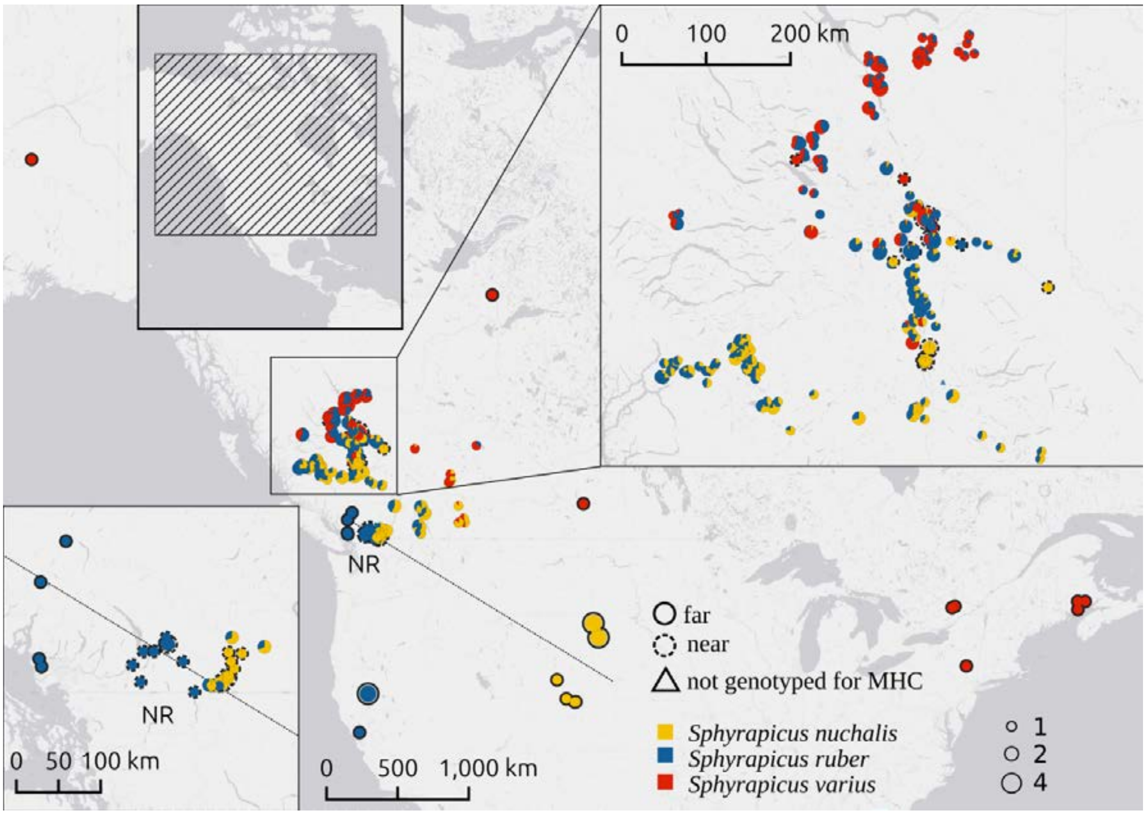

Figure S10 – *Sphyrapicus*. Sampling, genome-wide ancestry, classification of populations into parapatry and allopatry, and designated transect.

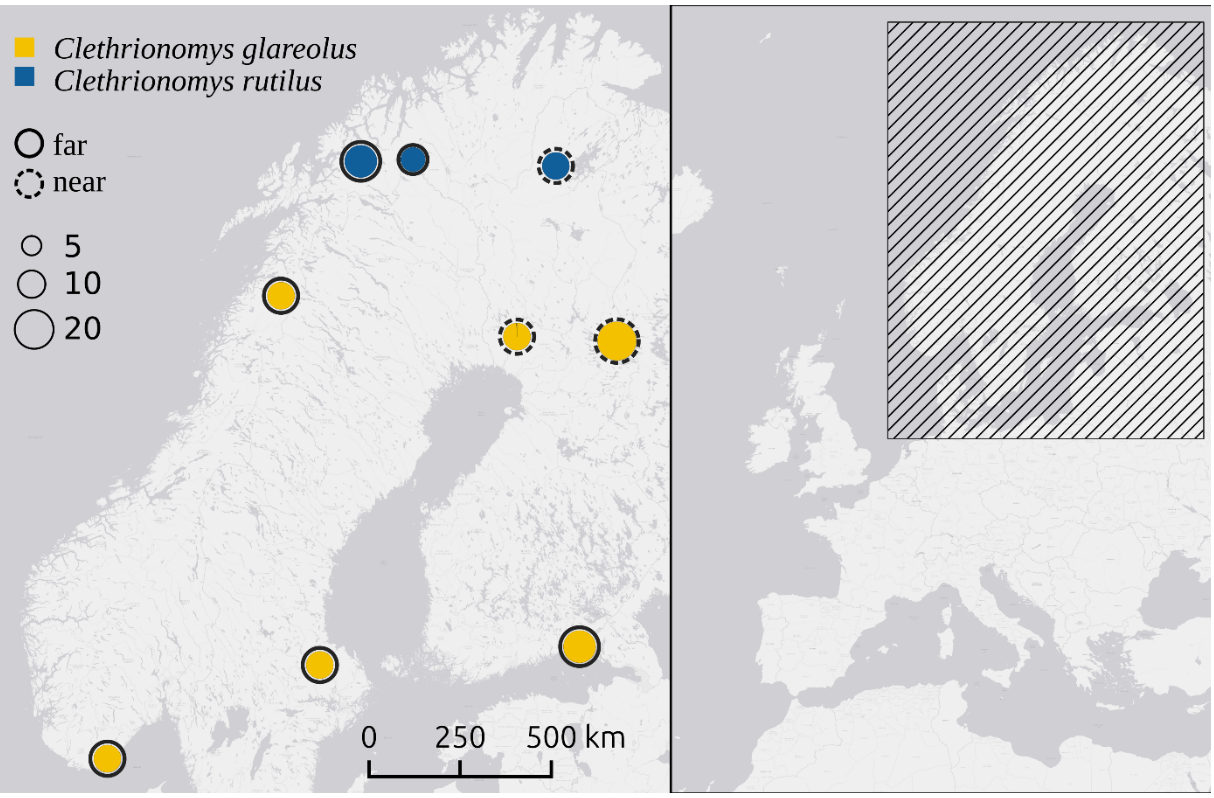

Figure S11 – *Clethrionomys*. Sampling, genome-wide ancestry, and classification of populations into parapatry and allopatry.
